# Peptidoglycan Endopeptidase Spr of Uropathogenic *Escherichia coli* Contributes to Kidney Infections and Competitive Fitness During Bladder Colonization

**DOI:** 10.3389/fmicb.2020.586214

**Published:** 2020-12-16

**Authors:** Wen-Chun Huang, Masayuki Hashimoto, Yu-Ling Shih, Chia-Ching Wu, Mei-Feng Lee, Ya-Lei Chen, Jiunn-Jong Wu, Ming-Cheng Wang, Wei-Hung Lin, Ming-Yuan Hong, Ching-Hao Teng

**Affiliations:** ^1^ Institute of Molecular Medicine, College of Medicine, National Cheng Kung University, Tainan, Taiwan; ^2^ Institute of Basic Medical Sciences, College of Medicine, National Cheng Kung University, Tainan, Taiwan; ^3^ Center of Infectious Disease and Signaling Research, National Cheng Kung University, Tainan, Taiwan; ^4^ Institute of Biological Chemistry, Academia Sinica, Taipei, Taiwan; ^5^ Department of Cell Biology and Anatomy, College of Medicine, National Cheng Kung University, Tainan, Taiwan; ^6^ Department of Biotechnology, National Kaohsiung Normal University, Kaohsiung, Taiwan; ^7^ Department of Biotechnology and Laboratory Science in Medicine, School of Biomedical Science and Engineering, National Yang Ming University, Taipei, Taiwan; ^8^ Division of Nephrology, Department of Internal Medicine, National Cheng Kung University Hospital, College of Medicine, National Cheng Kung University, Tainan, Taiwan; ^9^ Department of Emergency Medicine, National Cheng Kung University Hospital, College of Medicine, National Cheng Kung University, Tainan, Taiwan

**Keywords:** Spr, MepS, urinary tract infections, bacteremia, motility, flagella, the complement system

## Abstract

Uropathogenic *E* *scherichia coli* (UPEC) is the most common pathogen of urinary tract infections (UTIs). Antibiotic therapy is the conventional measure to manage such infections. However, the rapid emergence of antibiotic resistance has reduced the efficacy of antibiotic treatment. Given that the bacterial factors required for the full virulence of the pathogens are potential therapeutic targets, identifying such factors may facilitate the development of novel therapeutic strategies against UPEC UTIs. The peptidoglycan (PG) endopeptidase Spr (also named MepS) is required for PG biogenesis in *E. coli*. In the present study, we found that Spr deficiency attenuated the ability of UPEC to infect kidneys and induced a fitness defect during bladder colonization in a mouse model of UTI. Based on the liquid chromatography (LC)/mass spectrometry (MS)/MS analysis of the bacterial envelope, *spr* deletion changed the levels of some envelope-associated proteins, suggesting that Spr deficiency interfere with the components of the bacterial structure. Among the proteins, FliC was significantly downregulated in the *spr* mutant, which is resulted in reduced motility. Lack of Spr might hinder the function of the flagellar transcriptional factor FlhDC to decrease FliC expression. The motility downregulation contributed to the reduced fitness in urinary tract colonization. Additionally, *spr* deletion compromised the ability of UPEC to evade complement-mediated attack and to resist intracellular killing of phagocytes, consequently decreasing UPEC bloodstream survival. Spr deficiency also interfered with the UPEC morphological switch from bacillary to filamentous shapes during UTI. It is known that bacterial filamentation protects UPEC from phagocytosis by phagocytes. In conclusion, Spr deficiency was shown to compromise multiple virulence properties of UPEC, leading to attenuation of the pathogen in urinary tract colonization and bloodstream survival. These findings indicate that Spr is a potential antimicrobial target for further studies attempting to develop novel strategies in managing UPEC UTIs.

## Introduction

Uropathogenic *Escherichia coli* (UPEC) are responsible for approximately 75 and 65% of community- and hospital-acquired urinary tract infections (UTIs; Foxman, 2010). The diseases result in significant morbidity and healthcare costs (Foxman, 2003). Antibiotic therapy is the most common treatment for bacterial infections. However, the emergence of multiple antibiotic-resistant strains significantly interferes with the efficacy of the treatment, which has posed a substantial threat to public health worldwide. Bacterial factors required for maintaining the full virulence and optimal fitness of the pathogens are potential antimicrobial targets against the infections (Subashchandrabose and Mobley, 2015). In addition, UPEC strains are characterized by substantial diversity and as a result, multiple different classical virulence factors are not conserved across strains (Takahashi et al., 2006; Mao et al., 2012; Sintsova et al., 2019). Thus, identifying fitness and virulence factors that are commonly present in UPEC strains may facilitate the development of novel therapeutic strategies that can be widely used to treat infections caused by different UPEC strains.

Spr (also named MepS) is a bacterial D,D-endopeptidase, which commonly exists in *E. coli*, and is involved in peptidoglycan (PG) biogenesis. It cleaves peptide cross-bridges between glycan chains to facilitate the incorporation of newly synthesized glycan strands into the growing PG sacculus in the bacterial envelope (Singh et al., 2012). *Escherichia coli* without *spr* fails to resist osmotic shock at high temperature (Hara et al., 1996). Additionally, disruption of *spr* sensitizes *Salmonella enterica* serovar Typhimurium to the glycopeptide antibiotic vancomycin (Vesto et al., 2018). These findings suggest that Spr-deficiency may interfere with the integrity of the bacterial envelope, and thus attenuate bacterial resistance against harsh environments. Given that the envelope is the frontline of UPEC to interact with hosts during infections, it is worth investigating whether inhibition of Spr impairs the ability of the bacteria to invade the urinary tract.

Uropathogenic *Escherichia coli* can infect urinary tracts through ascending or descending pathways. The majority of UTIs are caused through the ascending pathway, in which bacteria gain access to urinary tracts through the urethra. From the urethra, the bacteria ascend to the bladder to cause cystitis, which may be followed by ascension to the kidneys to cause pyelonephritis. In some severe cases of pyelonephritis, UPEC can enter the bloodstream to cause bacteremia and, occasionally, urosepsis (Bien et al., 2012). Rarely, some UTIs occur by way of the descending pathway. Descending infections are the result of hematogenous spread of bacteria from a primary source located elsewhere in the body.

To achieve UTIs, UPEC requires multiple virulence properties to be able to disseminate within and colonize the urinary tract, and to tackle the host immune system. The motility of UPEC contributes to bacterial colonization and dissemination within urinary tracts (Lane et al., 2005, 2007a; Wright et al., 2005). The bacterial ability to bind to epithelial cells lining the bladder is required for the pathogen to avoid rapid clearance with bulk flow of urine (Bower et al., 2005). In addition, the competence of UPEC to resist or evade immune system-mediated killing is critical for the pathogen survival during the process of gaining access to and in urinary tracts (Hunstad and Justice, 2010; Miajlovic and Smith, 2014; Olson and Hunstad, 2016). In the present study, we investigated whether deficiency of Spr impaired these virulence properties.

The bacterial motility of UPEC is mainly attributed to the propelling force of flagella. Flagellar expression is regulated by a three-tier hierarchical cascade of the flagellar regulon (Soutourina and Bertin, 2003; Wang et al., 2006; Terashima et al., 2008). At the top of this cascade (class 1) is the master operon, *flhDC*, which encodes the subunits (FlhD and FlhC) of functional heterohexamer (FlhD_4_C_2_) that acts as an essential transcription activator of the class 2 genes (Takaya et al., 2006). The class 2 genes encode the flagellar basal body and hook proteins, as well as the flagellum-specific sigma factor σ^28^ (FliA) and anti-σ^28^ factor (FlgM). FliA is required for the transcription of class 3 genes (Ohnishi et al., 1990). The class 3 genes encode flagellin (FliC), components of the motor, and chemotaxis-related regulatory factors (Terashima et al., 2008). Since FlhDC is essential for the transcription of all flagellar genes, the cellular levels of FlhDC are tightly modulated in a transcriptional (Soutourina and Bertin, 2003; Pruss, 2017), posttranscriptional (Wei et al., 2001; De Lay and Gottesman, 2012; Thomason et al., 2012; Mika and Hengge, 2013; Yakhnin et al., 2013), and posttranslational manner (Tomoyasu et al., 2003; Takaya et al., 2006, 2012; Kitagawa et al., 2011). It has been shown that the expression of flagella can be regulated by extracytoplasmic stress signaling systems, such as two-component systems and heat shock response (Li et al., 1993; Shi et al., 1993; Alteri et al., 2011; Pruss, 2017; Huang et al., 2020b) that can sense alternation of the bacterial envelope and external environmental stimuli (Vianney et al., 2005; Kumar et al., 2012; Evans et al., 2013).

The morphological switch of UPEC from a bacillary to an elongated filamentous shape is proposed to be one of the bacterial strategies to subvert the innate immunity of hosts during UTIs (Justice et al., 2008; Horvath et al., 2011). The filamentation of UPEC occurs after the pathogen interacts with the bladder epithelium during UTIs (Justice et al., 2008). In the bladder, the bacteria can adhere to and then invade the bladder epithelial cells (Justice et al., 2008). After invasion, UPEC replicates and forms an intracellular bacterial community (IBC) within the host cells. During the maturation of IBC, a subpopulation of bacteria stops cell division, resulting in elongated filamentous bacteria. When this intracellular bacterial burden leads to host cell death and ultimately breaks out of the host cell, the filamentous bacteria are released into the bladder lumen, where UPEC may encounter host phagocytes. Filamentation promotes UPEC resistance to phagocytosis (Horvath et al., 2011).

In this study, we found that the loss of *spr* attenuates the ability of UPEC to infect kidneys and decreases the competitive fitness in bladders and the bloodstream. The *spr* deletion significantly decreased motility, formation of filamentous cells, and resistance to innate immunity, which may explain its reduced capacity to survive in the urinary tract and bloodstream.

## Materials and Methods

### Ethics Approval Statement

All animal studies were carried out according to the guideline by Council of Agriculture Executive Yuan Guideline for the Care and Use of Laboratory Animals, Republic of China. All of the animal experimental procedures were reviewed and approved by the Institutional Animal Care and Use Committee (IACUC) of National Cheng Kung University, Tainan City, Taiwan (approval number: 105175 and 107175). The procedures for collecting human serum and urine samples were approved by the Institutional Reviewer Board (IRB) of National Cheng Kung University Hospital, Tainan City, Taiwan (no. ER-98-143 and B-ER-108-308). The informed consents were obtained from healthy volunteers according to the relevant guideline of the IRB.

### Bacterial Strains, Plasmids and Growth Condition

The bacterial strains and plasmids used in this study are shown in Table 1. Bacteria were grown in Luria Bertani (LB) broth at 37°C for 16 h unless otherwise indicated and were stored in LB with a final concentration of 15% glycerol at −80°C. Antibiotics were used at the following concentrations: ampicillin (100 μg/ml), chloramphenicol (15 μg/ml), and spectinomycin (100 μg/ml). The procedures to manipulate pathogenic *E. coli* at a biosafety level 2 (BSL-2) laboratory were reviewed and approved by the Biosafety and Radiation Safety Division, Center for Occupational safety and Health and Environmental Protection of National Cheng Kung University, Tainan City, Taiwan.

**Table 1:** *E. coli* strains and plasmids used in this study.

Strain or plasmid	Relevant information	AR marker^*^	Reference
Strain			
WT-UTI89	UTI89 isolated from the urine of a patient with cystitis	-	Chen et al., 2006
Δ*spr*-UTI89	UTI89 with a *spr* deletion	Cm	This study
Δ*lacZ*-UTI89	UTI89 with a *lacZ* deletion	-	Huang et al., 2020b
*lacZ*::*spr*Δ*spr*-UTI89	Δ*spr*-UTI89 with complementary *spr* on the *lacZ* gene chromosomal locus	-	This study
Spr-C68A-UTI89	UTI89 with the *spr* gene containing the catalytic site Cys68 replaced by Ala resulting in inactivation of the endopeptidase activity	Cm	This study
Δ*flhDC*-UTI89	UTI89 with *flhDC* deletion	Cm	This study
Δ*flhDC*Δ*spr*-UTI89	UTI89 with *flhDC* and *spr* deletions	Cm	This study
Δ*spr*Δ*lacZ*-UTI89	UTI89 with *spr* and *lacZ* deletions	Cm	This study
Δ*spr*Δ*flhDC*Δ*lacZ*-UTI89	UTI89 with *spr*, *flhDC* and *lacZ* deletions	Cm	This study
*flhD-lacZ*-UTI89	UTI89 with *flhD* promoter-*lacZ* transcriptional fusion on chromosome	Cm	This study
*flhD-lacZ*-Δ*spr*-UTI89	Δ*spr*-UTI89 with *flhD* promoter-*lacZ* transcriptional fusion on chromosome	Cm	This study
*fliA-lacZ*-UTI89	UTI89 with *fliA* promoter-*lacZ* transcriptional fusion on chromosome	Cm	This study
*fliA-lacZ*-Δ*spr*-UTI89	Δ*spr*-UTI89 with *fliA* promoter-*lacZ* transcriptional fusion on chromosome	Cm	This study
*flhA-lacZ*-UTI89	UTI89 with *flhA* promoter-*lacZ* transcriptional fusion on chromosome	Cm	This study
*flhA-lacZ*-Δ*spr*-UTI89	Δ*spr*-UTI89 with *flhA* promoter-*lacZ* transcriptional fusion on chromosome	Cm	This study
*fliM-lacZ*-UTI89	UTI89 with *fliM* promoter-*lacZ* transcriptional fusion on chromosome	Cm	This study
*fliM-lacZ*-Δ*spr*-UTI89	Δ*spr*-UTI89 with *fliM* promoter-*lacZ* transcriptional fusion on chromosome	Cm	This study
*fliT-lacZ*-UTI89	UTI89 with *fliT* promoter-*lacZ* transcriptional fusion on chromosome	Cm	This study
*fliT-lacZ*-Δ*spr*-UTI89	Δ*spr*-UTI89 with *fliT* promoter-*lacZ* transcriptional fusion on chromosome	Cm	This study
*fliC-lacZ*-UTI89	UTI89 with *fliC* promoter-*lacZ* transcriptional fusion on chromosome	Cm	This study
*fliC-lacZ*-Δ*spr*-UTI89	Δ*spr*-UTI89 with *fliC* promoter-*lacZ* transcriptional fusion on chromosome	Cm	This study
Plasmid			
pUC18-FlhDC	pUC18 harboring a sequence encoding the N-terminally HA-tagged FlhD and C-terminally His_6_-tagged FlhC that were under control of its own promoter	Amp	This study
pUC19	Expression plasmid containing the *lac* promoter	Amp	NEB
pFlhDC	pUC19 harboring a sequence encoding the N-terminally HA-tagged FlhD and C-terminally His_6_-tagged FlhC that were under control of *lac* promoter	Amp	Huang et al., 2020b
pFPV25.1	GFP expressing plasmid	Amp	Valdivia and Falkow, 1996
pKD3	Template plasmid for FRT-flanked *cat* cassette	Cm	Datsenko and Wanner, 2000
pKD46	λ Red recombinase expression plasmid	Amp	Datsenko and Wanner, 2000

* AR marker, antibiotic resistance marker; Cm, chloramphenicol; Amp, ampicillin.

### Human Sera and Urine

The normal human serum (NHS) used in this study was pooled from the serum of seven healthy adults and stored in aliquots at −80°C. Heat-inactivated NHS (HI-NHS) was prepared by heating NHS at 56°C for 30 min. Human urine was pooled from five healthy adults. The urine was filtered with 0.45-μm and then 0.22-μm pore-size filters and briefly heated to 70°C thereafter to inactivate potential organisms and immune components. The pooled urine was stored at 4°C until use. Urine concentration was measured as urine specific gravity (USG). The USG of the pooled urine samples used in the study was 1.030.

### Mutant Construction

The gene deletion mutants, flagellar promoter-*lacZ* fusion strains, *spr* complementary strain, and *spr* point mutation strain were constructed according to the PCR-based gene inactivation method previously described by Datsenko and Wanner (2000). PCR products containing chloramphenicol-resistance cassettes flanked by approximately 40 or 500 bp of homology to the upstream and downstream regions of target genes were generated by overlap extension PCR (Nelson and Fitch, 2011) using primers shown in Supplementary Table S1. The homology upstream and downstream regions of target genes were amplified from WT-UTI89. The chloramphenicol-resistance cassette was amplified from pKD3. These PCR products were electroporated into UTI89 strains containing pKD46. The resulting bacterial strains were selected on chloramphenicol plates. After gene deletion or insertion, the size of the original gene locus changes. Therefore we further confirm the deletion and insertion mutants by determining the sizes of the target loci with PCR amplification of the modified regions. To confirm mutants with point mutations, sequencing of the related regions was performed.

### Construction of Recombinant Plasmid pUC18-FlhDC

The DNA fragment containing the *flhDC* promoter region and the DNA fragment encoding the N-terminally HA-tagged FlhD and C-terminally His_6_-tagged FlhC were amplified from the UTI89 genome by PCR with the primer set, *flhDC* promoter-F and *flhDC* promoter-R, and the primer set, HA-*flhD*-F and *flhC*-R, respectively (Supplementary Table S1). The amplified fragments were cleaned using a DNA extraction kit (ProTech, Taiwan) and then fused by overlap extension PCR (Nelson and Fitch, 2011). The generated PCR product was digested with *HindIII* and *BamHI* and then cloned into the digested plasmid vector pUC18.

### Mouse Infection Models

The mouse model of UTI was performed as described previously (Lane et al., 2005; Huang et al., 2020b) with some modifications. In the co-infection experiments, 8-week-old female C3H/HeN mice were inoculated transurethrally with a 1:1 mixture of two indicated bacterial strains (5 × 10^7^ CFU/strain). At 48 h postinfection, the bladders and kidneys were collected, weighed, and homogenized in sterile culture tubes containing 3 ml of normal saline. Bacterial counts were determined by plating the homogenates on LB agar plates containing IPTG and X-gal to differentiate between the two strains. The strains with and without *lacZ* showed blue and white colonies on the plates, respectively. In the independent infection experiments, 1 × 10^8^ CFU of WT-UTI89, ∆*spr*-UTI89, or *lacZ*::*spr*∆*spr*-UTI89 were inoculated into mice. At 48 h postinfection, the bacterial counts in the bladders and kidneys were determined.

In addition, to investigate the filamentation of invading UPEC in the bladder, UPEC strains with and without *spr* deletion were independently inoculated into the bladder of mice. At 12 h postinoculation, urine samples were collected and fixed with 10% paraformaldehyde followed by DAPI staining for further microscopic inspection. The length of the bacteria was determined by ImageJ (National Institutes of Health; Bethesda, MD, United States).

The co-infection mouse model of bacteremia was performed as described previously (Huang et al., 2020a) with some modification. Eight-week-old BALB/c mice were intraperitoneally inoculated with a 1:1 mixture of two indicated bacterial strains (5 × 10^6^ CFU/strain). After 6 h, blood samples were collected from mice. Bacterial counts in the blood were determined by plating the blood samples on LB agar plates with and without chloramphenicol to differentiate the *E. coli* strains (Table 1).

### Liquid Chromatography-Tandem Mass Spectrometry (LC/MS/MS) Analyses of Bacterial Proteins

The bacterial proteins for the LC/MS/MS analyses were extracted from 16-h cultures of WT-UTI89 and Δ*spr*-UTI89. Three independent cultures for each strain were prepared from the analyses. The bacteria were collected from the cultures by centrifugation and resuspended in Tris-HCl buffer (50 mM, pH 7.5). The bacterial suspensions were subjected to French press at 8,000 lb/in^2^ followed by centrifugation at 6,000 *g* for 10 min to pellet and remove unbroken cells. The resulting supernatants were centrifuged at 100,000 *g* at 4°C for 1 h to pellet envelope fractions. The envelope fractions were resuspended in Tris-HCl buffer, and then subjected to 12.5% SDS-PAGE to separate the proteins in the samples. The gel lane of each sample was cut into two slices and was subjected to in-gel digestion with trypsin followed by protein identification with the Thermo LTQ-Orbitrap Velos system. The MS/MS spectra were searched against *Escherichia coli* SwissProt 2018_01 (556,568 sequences; 199,530,821 residues) using Proteome Discoverer 2.2 (Thermo Fisher, United Kingdom). The LC/MS/MS raw data are shown in Supplementary Table S2. Subsequently, the protein identifications with two peptides in at least one of the samples were retained. The proteins exhibiting at least a 2-fold significance difference between WT-UTI89 and Δ*spr*-UTI89 are shown in Table 2.

**Table 2:** Identification of altered envelope proteins by liquid chromatography-tandem mass spectrometry.

Protein name	Fold change^*^	P value	Location
Downregulated protein in Δ*spr*-UTI89			
Acriflavine resistance protein AcrE	−100	0.009	IM
LPS export system protein LptC	−17.99	0.029	IM
Flagellin/FliC	−4.99	0.014	E
Probable TonB-dependent receptor YncD	−2.55	0.015	OM
G3P-binding periplasmic protein UgpB	−2.34	0.022	P
Upregulated protein in Δ*spr*-UTI89			
NfeD-like family protein YbbJ	+2.15	0.032	IM
Endolytic murein transglycosylase YceG	+2.25	0.032	IM
Tail anchored inner membrane protein ElaB	+2.41	0.038	IM
Outer membrane lipoprotein SlyB	+2.42	0.020	OM
Uncharacterized protein YhcB	+2.6	0.030	IM
Uncharacterized protein YniB	+3.96	0.025	IM
DUF445 domain-containing protein YjiN	+5.96	0.032	IM
Heat shock protein HslJ	+14.91	0.022	M

* -indicates that the protein was downregulated in Δspr-UTI89 compared to WT-UTI89.

+indicates that the protein was upregulated in Δspr-UTI89 compared to WT-UTI89. IM, inner membrane; M, membrane; E, extracellular space; OM, outer membrane; P, periplasm.

### Western Blot Analysis

The western blot analysis was performed as described previously (Teng et al., 2010) with some modification. Equal amount of total bacterial proteins were separated by SDS-PAGE, and then transferred to PVDF membranes (Pall Corporation). The rabbit polyclonal antisera against flagellin (anti-H7, Becton Dickinson, Sparks, MD, United States) was used as the primary antibody (1:10,000 dilution), and the goat anti-rabbit horseradish peroxidase (HRP)-conjugated immunoglobulin G (IgG) antibodies (1:10,000 dilution; KPL, Gaithersburg, MD) were used as the secondary antibodies to detect the protein. The mouse antiserum against OmpA, anti-HA antibody and anti-His_6_ antibody (Sigma-Aldrich, St. Louis, MO, United States) were used as the primary antibodies (1:10,000 dilution), and the goat anti-mouse HRP-conjugated IgG antibodies (1:10,000 dilution; KPL, Gaithersburg, MD) were used as the secondary antibody.

### Motility Assay

Bacterial strains were stab-inoculated into 0.3% agar plates and incubated at 37°C for 8 h (Teng et al., 2010). The diameter of motility was measured from experiments in triplicate and presented as the means ± standard deviations.

### RNA Isolation and Real-Time PCR

RNA isolation and qPCR assays were carried out as described previously (Huang et al., 2020b). The expression levels of the genes were normalized to those of *gyrB* and are presented as relative rates compared to the activity of the wild-type strain. The primers used for these assays are shown in Supplementary Table S1.

### Survival in SDS Condition Assay

Bacteria from 16-h cultures in LB medium were diluted 1:100 into 2 ml fresh LB medium without sodium dodecyl sulfate (SDS), or with 5% SDS. The bacteria were grown to 3 h at 37°C. The bacterial counts were determined by plating on LB agar. The levels of bacterial survival are presented as relative survival rates compared to those of WT-UTI89.

### Survival of the Low pH Condition Assay

Bacteria from 16-h cultures were diluted 1:100 in 2 ml LB medium with pH 7.5 or pH 4.5 and were grown to 2 h at 37°C. The bacterial counts were determined by plating on LB agar. The levels of bacterial survival are presented as relative survival rates compared to those of WT-UTI89.

### Macrophage Survival Assay

To assess the contribution of Spr in the intracellular survival of UPEC in phagocytes, UTI89 strains with or without *spr* were incubated with the macrophage cell line RAW264.7, respectively for 30 min. The multiplicity of infection (MOI) was 10. The infected cells were treated with 100 μg/ml gentamicin and incubated for 15 min to kill extracellular bacteria. Then, the cell culture was replaced with medium containing a lower concentration of gentamicin (10 μg/ml) for the remainder of the experiment. After incubation in low gentamicin medium for 0 and 24 h, the bacterial counts in RAW264.7 macrophages were determined. The levels of bacterial survival are presented as relative survival rates compared to those of WT-UTI89.

### Serum Survival Assay

Each bacterial strain (1 × 10^6^ CFU) was independently incubated in 100 μl of 30% pooled NHS or heat-inactivated human serum (HI-NHS) diluted with PBS. To inactivate the complement system, HI-NHS was prepared by heating at 56°C for 30 min. After different lengths of time, the survival of *E. coli* strains was determined. The survival rates were calculated by the ratio of the bacterial counts to those of the original inocula at different time points.

### Flow Cytometry Analysis

*Escherichia coli* (3 × 10^7^ CFU) were incubated at 37°C in 100 μl of 30% NHS in veronal buffer (Lonza, Walkersville, MD, United States) for 1, 5, and 15 min. After three washes with PBS, the bacteria were incubated with antibodies against the FITC-conjugated anti-C3b antibody (Abcam, Cambridge, MA, United States) at room temperature for 1 h followed by three washes with PBS. The surface deposition of the C3b molecule was analyzed with a FACSCalibur flow cytometer (Becton Dickinson, San Jose, CA, United States).

### Flow Chamber-Based Cell Infection Model

Flow chamber (FC) bladder cell infection was performed as described previously (Andersen et al., 2012; Khandige et al., 2016) with some modification. The dimensions of the FCs were 0.15 cm (height) × 0.2 cm (width) × 2 cm (length). Flow medium was brought to and moved through the opens on the top of both ends of the chamber. The bladder epithelial cell line 5,637 was seeded and grown to a confluent monolayer on the bottom of the chamber in RPMI containing 10% FBS and 1% penicillin and streptomycin (Pen-Strep; Invitrogen) under static conditions. The cultures were grown under a continuous flow of Epilife medium (Invitrogen) with 1% human keratinocyte growth medium (HKGM) and 1% Pen-Strep (0.18 ml/h). The antibiotics in the medium were removed 1 h before bacterial infection. Then, the cells in the chamber were infected by shifting to a flow of GFP-expressing bacterial suspension in PBS (1 × 10^5^ CFU/μl) for 20 min at a rate of 20 μl/min. To allow bacterial invasion and replication within the cells, the flow was shifted to Epilife medium supplemented with 0.5% peptone and 0.5% glucose at a rate of 0.18 ml/min. After 6 h of incubation, gentamicin and amikacin were added to the flow medium to a final concentration of 100 μg/ml to kill the bacteria remaining outside the epithelial cells. After 2 h of antibiotic incubation, the FC culture was shifted to a flow (0.18 ml/min) of human urine to mimic the UPEC-bladder epithelium interaction during UTIs. After 24 h of incubation, the bacteria in the FC system were assessed by fluorescence microscopy. The length of the bacteria was determined by ImageJ (National Institutes of Health; Bethesda, MD, United States).

### Association and Invasion Assay

The bladder epithelial cell lines 5637 and T24 were seeded into 24-well plates in RPMI-1640 media with 10% FBS and 1% penicillin-streptomycin and grown to a monolayer. Before 2 h of infection, the culture medium was replaced with fresh medium without 1% penicillin-streptomycin. The cells were infected with a MOI of 10 of the wild-type UTI89 or the *spr* mutant, centrifuged at 600 *g* for 5 min to synchronize bacteria-host cell contact, and incubated at 37°C for 1.5 h. The cells were washed once with PBS and then incubated with medium containing gentamicin (100 μg/ml) for 1 h to kill extracellular bacteria and then washed three times with PBS. The cells were lysed by incubation with sterile water at 4°C for 30 min and plated on LB plates. The resulting colonies were counted to determine intracellular bacteria. The invasion frequencies were calculated by dividing the number of internalized bacteria by the number of the original inoculum. The results were presented as relative invasiveness, the percent invasion compared to the invasion frequency of the wild-type UTI89, which was arbitrarily set at 100%. Association assays were performed as described for the invasion assay above, except that the gentamicin treatment step was omitted.

### Statistical Analysis

Coinfection experiments of animal experiments were analyzed by using a nonparametric Wilcoxon matched-pair test while the independent infection experiments of animal experiments were analyzed by using a nonparametric Mann-Whitney test (Lane et al., 2005). Other experiments were performed to determine significant differences by two-tailed Student’s *t* test. A value of *p* < 0.05 was considered to be significant (GraphPad Prism 7; GraphPad Software Inc., La Jolla, CA, United States; ^*^ *p* < 0.05; ^**^ *p* < 0.01; ^***^ *p* < 0.001).

## Results

### Deletion of *spr* Decreases the Competitive Fitness of UPEC During UTIs and Attenuates the Ability to Infect Kidneys

**Figure 1:**
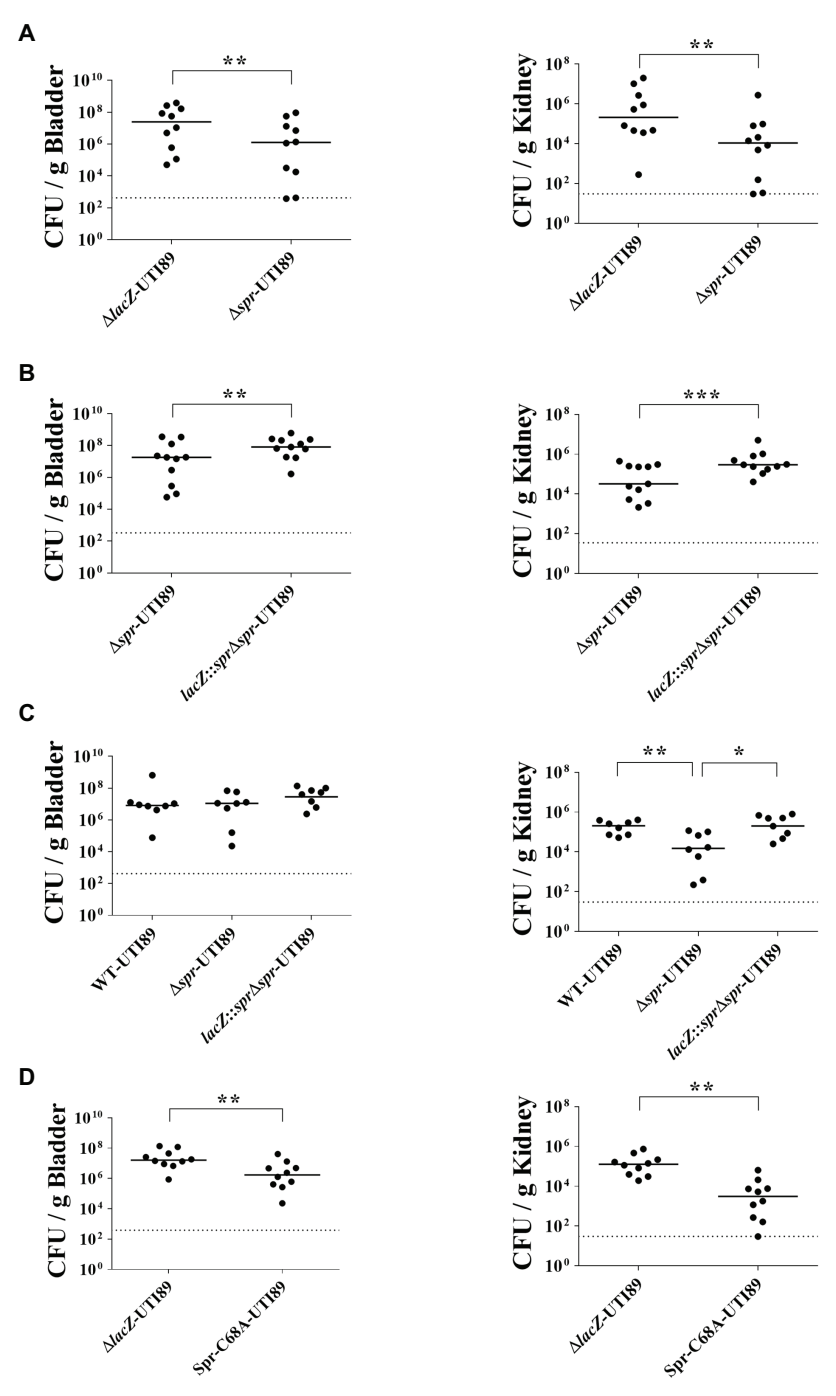
Role of *spr* in Uropathogenic *Escherichia coli* (UPEC)-induced urinary tract infections (UTIs). **(A)** The bacterial counts of ∆*lacZ*-UTI89 and ∆*spr*-UTI89 in the bladders and kidneys at 48 h after transurethral co-inoculation with equal amounts of these strains (5 × 10^7^ CFU/mouse for each strain). Animal numbers *N* = 10. **(B)** The bacterial counts of *lacZ*::*spr*∆*spr*-UTI89 and ∆*spr*-UTI89 in the bladders and kidneys at 48 h after transurethral co-inoculation with equal amounts of these strains (5 × 10^7^ CFU/mouse for each strain). *N* = 11. **(C)** The bacterial counts of WT-UTI89, ∆*spr*-UTI89, and *lacZ*::*spr*∆*spr*-UTI89 in the bladders and kidneys at 48 h after transurethral inoculation with these strains (1 × 10^8^ CFU/mouse), respectively. *N* = 8 for each strains. **(D)** The bacterial counts of Spr-C68A-UTI89 and ∆*spr*-UTI89 in the bladders and kidneys at 48 h after transurethral co-inoculation with equal amounts of these strains (5 × 10^7^ CFU/mouse for each strain). N = 10. The horizontal bars represent the median values. The dotted line represents the limit of detection. ^*^ *p* < 0.05; ^**^ *p* < 0.01; ^***^ *p* < 0.001.

To investigate the role of *spr* in UTIs, equal numbers of the *spr* mutant of the UPEC strain UTI89 (∆*spr*-UTI89) and the otherwise wild-type *lacZ* mutant (∆*lacZ*-UTI89) were coinoculated into the urinary tract of C3H/HeN mice. Their bacterial counts in the bladders and kidneys were determined at 48 h postinoculation (*lacZ* deletion did not affect UPEC colonization on the urinary tract; Supplementary Figure S1). The bacterial burdens in the bladder and kidney of ∆*spr*-UTI89 were significantly lower than those of ∆*lacZ*-UTI89 (Figure 1A).

To confirm whether the decreased bacterial counts of ∆*spr*-UTI89 are due to the loss of *spr* and not a polar effect, we performed complementation assays of this mutant. A functional copy of *spr* was inserted into the chromosomal *lacZ* locus of ∆*spr*-UTI89, resulting in the complement strain *lacZ*::*spr*∆*spr*-UTI89 (Table 1). As shown in Figure 1B, complementation with the *spr* gene significantly increased the *spr* mutants’ counts in the bladder and kidney. These findings suggested that Spr is required for maintaining the competitive fitness of UPEC during UTIs.

To determine whether Spr contributes to UTIs in independent infection experiments, WT-UTI89, ∆*spr*-UTI89, and *lacZ*::*spr*∆*spr*-UTI89 were independently inoculated into the urinary tract of animals. As shown in Figure 1C, at 48 h postinoculation, ∆*spr*-UTI89 exhibited significantly lower bacterial counts in kidneys in comparison to WT-UTI89 and *lacZ*::*spr*∆*spr*-UTI89, while the strains showed no significant difference in bacterial counts in bladders. These results suggested that Spr deficiency attenuate the ability of UPEC to infect kidneys.

We further investigated whether deficiency of the Spr endopeptidase function is responsible for the reduced fitness of ∆*spr*-UTI89 during UTIs. Because the Spr residue Cys-68 is essential for the catalytic activity of this endopeptidase, Spr with an alanine substitution at this position (Spr-C68A) has no endopeptidase activity (Singh et al., 2012). An UTI89 strain (Spr-C68A-UTI89), which expresses Spr-C68A instead of wild-type Spr, was constructed, and its ability to induce UTIs was evaluated. As shown in Figure 1D, Spr-C68A-UTI89 exhibited a lower ability to colonize the urinary tract compared to the otherwise wild-type strain, ∆*lacZ*-UTI89, indicating that the endopeptidase function of Spr is required for the competitive fitness of UPEC *in vivo*.

To investigate whether *spr* deletion induces a general fitness defect of growth, the growth curves of WT-UTI89 and ∆*spr*-UTI89 in LB and M9 media and urine were determined with co-culture and/or independent culture experiments. The strains showed similar growth curves in the experiments (Supplementary Figure S2), suggesting that *spr* deletion does not interfere with basic growth fitness of UPEC and that the fitness defect of the *spr* mutant is specific to UTIs.

### Deletion of *spr* Changes the Expression of Envelope-Associated Proteins

Peptidoglycan is one of the major structures of the *E. coli* cell envelope that also contains the outer membrane (OM) and inner membrane (IM). The envelope is the frontline of invading bacteria encountering hostile host environments during infections. To assess whether Spr deficiency affects the components of the envelope, the envelope fractions of WT-UTI89 and ∆*spr*-UTI89 were extracted and analyzed with liquid chromatography-tandem mass spectrometry (LC/MS/MS). We identified 13 proteins whose expression levels showed more than 2-fold significant differences between the strains (Table 2). Eight and five of the proteins were upregulated and downregulated due to the deletion of *spr*, respectively. The functions of some of these proteins are involved in bacterial motility (FliC; Lane et al., 2005), cell shape maintenance, envelope integrity (ElaB, SlyB, and YhcB; Plesa et al., 2006; Li et al., 2012; Guo et al., 2019), and peptidoglycan biogenesis (YceG; Yunck et al., 2016), suggesting that Spr deficiency may interfere with these functions. Since bacterial motility, morphological switching, and envelope integrity have been shown to be required for virulence of UPEC (Horvath et al., 2011; Flores-Mireles et al., 2015; Hews et al., 2019), the attenuated ability of ∆*spr*-UTI89 to cause UTI may be due to the altered envelope characteristics.

### *spr* Mutant of UPEC UTI89 Exhibits Decreased Motility and Flic Expression

**Figure 2:**
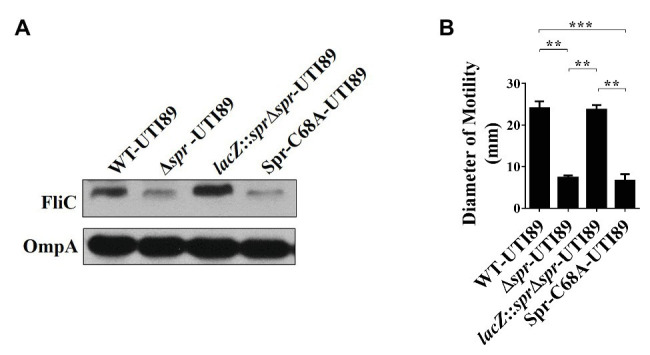
Effects of *spr* deletion on the FliC level and motility. **(A)** Western blot analyses of the FliC levels in the bacterial lysates of UTI89 strains. The levels of OmpA served as the loading control. **(B)** The motility of the UTI89 strains. The quantified motility of the indicated strains was derived from experiments performed in triplicate and is presented as the means ± standard deviations. ^**^ *p* < 0.01; ^***^ *p* < 0.001.

Since FliC is the major component of flagella and flagellum-mediated motility facilitates the fitness of UPEC during UTIs (Lane et al., 2005, 2007a; Wright et al., 2005), the downregulated FliC level in the envelope fractions may be responsible for the attenuated urovirulence of Δ*spr*-UTI89. Consistent with the above LC/MS/MS results, western blot analyses demonstrated that Δ*spr*-UTI89 showed a lower level of FliC than WT-UTI89 (Figure 2A). In addition, the mutant showed decreased motility compared to WT-UTI89 (Figure 2B). Complementation of Δ*spr*-UTI89 with the *spr* gene restored the expression level of FliC and motility (Figures 2A,B). These results suggested that Spr deficiency downregulates FliC expression and consequently decreases the motility of the UPEC *spr* mutant.

Additionally, we examined whether the reduced motility of the *spr* mutant is due to the loss of Spr endopeptidase function. As shown in Figures 2A,B, Spr-C68A-UTI89 exhibited lower FliC expression and motility than WT-UTI89. The findings suggested that deficiency of the endopeptidase activity is responsible for the defective FliC expression and motility in the *spr* mutant.

### Defective Motility Is Responsible for the Defect of Δ*spr*-UTI89 in Urinary Tract Infection

**Figure 3:**
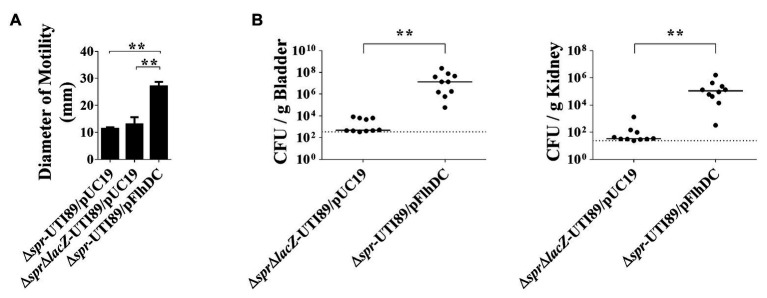
Contribution of bacterial motility to the abilities of the *spr*-deleted UPEC strains to cause UTIs. **(A)** The motilities of the UTI89 strains with or without overexpression of FlhDC. The quantification of motilities of the indicated strains. Each quantitative result was derived from experiments in triplicate and presented as the means ± standard deviations. **(B)** Effect of bacterial motility on the ability of the *spr* mutant strains to cause UTIs. *N* = 10. ^**^ *p* < 0.01.

To determine whether the decreased motility of Δ*spr*-UTI89 is responsible for the mutant’s attenuated ability to cause UTI, the plasmid pFlhDC, which harbors an *flhDC* and allowed overexpression of the flagellar master regulators, was introduced into Δ*spr*-UTI89, and the resulting strain was designated Δ*spr*-UTI89/pFlhDC. As shown in Figure 3A, the motility of Δ*spr*-UTI89/pFlhDC was significantly higher than those of the Δ*spr*-UTI89 and Δ*spr*Δ*lacZ*-UTI89 strains that harbor the empty vector pUC19 (Δ*spr*-UTI89/pUC19 and Δ*spr*Δ*lacZ*-UTI89/pUC19; Table 1). These results demonstrated that overexpressing *flhDC* is able to increase the motility of the *spr* mutant. We then evaluated whether the increased motility upregulates the ability of the *spr* mutant to colonize the urinary tracts. As shown in Figure 3B, after coinoculation into the urinary tracts of mice for 48 h, Δ*spr*-UTI89/pFlhDC showed higher levels of bacterial loads in the bladders and kidneys than Δ*spr*Δ*lacZ*-UTI89/pUC19. These results suggested that the reduced motility of the *spr* mutant attenuated its ability to colonize urinary tracts.

### Downregulated FliC and Motility Caused by Deletion of *spr* May Be Due to Suppression of the Function of the Master Regulator FlhDC

**Figure 4:**
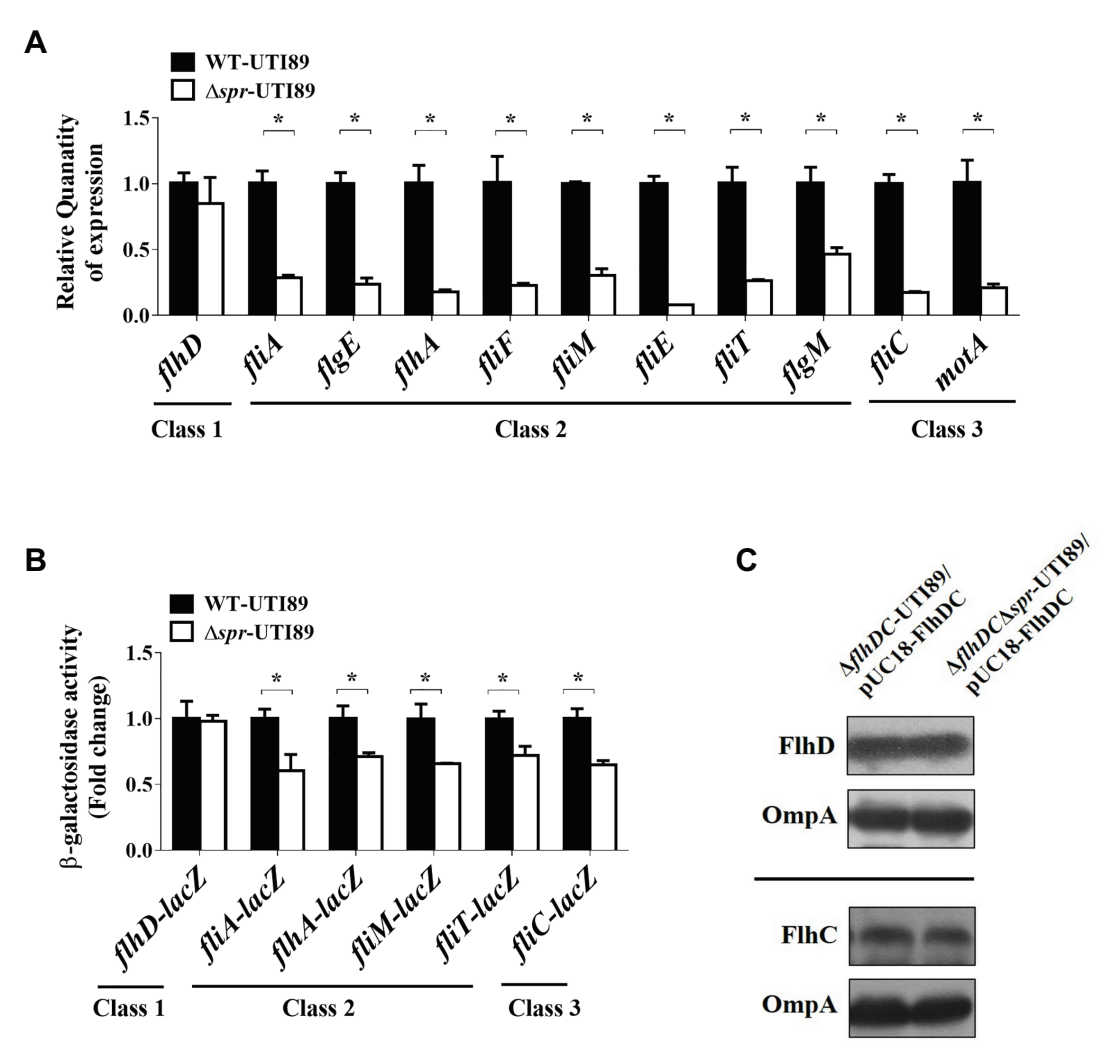
Effect of the *spr* mutation on flagella synthesis. **(A)** The mRNA expression of the flagellar regulon in WT-UTI89 and ∆*spr*-UTI89. The transcript levels of the class 1 gene *flhD*, the class 2 genes (*fliA, flgE, flhA, fliF, fliM, fliE, fliT* and *flgM*) and the class 3 genes (*fliC* and *motA*) were determined by real-time PCR (qPCR). The transcript levels of the genes in each strain, which were normalized to those of the housekeeping gene *gyrB*, were presented as the relative levels compared to those of WT-UTI89. The results were derived from experiments in triplicate and are shown as the means ± standard deviations. **(B)** The promoter activity of the flagellar regulon in WT-UTI89 and ∆*spr*-UTI89. The levels of β-galactosidase activity of *flhD*-*lacZ*, *fliA*-*lacZ*, *flhA*-*lacZ*, *fliM*-*lacZ*, *fliT*-*lacZ*, and *fliC*-*lacZ* operon fusion are presented as the relative levels compared to those of WT-UTI89. **(C)** The levels of HA-tagged FlhD and His_6_-tagged FlhC proteins in bacteria with and without *spr* were determined by western blot analysis with mouse anti-HA and anti-His_6_ antibodies, respectively. The levels of OmpA served as the loading control. ^*^ *p* < 0.05.

We further investigated the mechanism of how FliC levels are downregulated in the *spr* mutant. Because FliC expression is controlled by the transcriptional hierarchy of the flagellar regulon (Terashima et al., 2008), we assessed whether deletion of *spr* affected the transcription of this regulatory cascade. As shown in Figure 4A, Δ*spr*-UTI89 exhibited lower mRNA levels of the class 2 genes (*fliA*, *flgE*, *flhA*, *fliF*, *fliM*, *fliE*, *fliT*, and *flgM*) and class 3 genes (*fliC* and *motA*) compared to WT-UTI89. However, the mRNA levels of the class 1 gene *flhD* showed no significant difference between WT-UTI89 and Δ*spr*-UTI89.

We examined whether promoter activities of the class 2 and 3 genes are responsible for the lower mRNA levels in the *spr* mutant. To measure the activities of the promoters that drive the expression of the flagellar regulon genes, the promoter regions of the chromosomal *lacZ* genes of WT-UTI89 and Δ*spr*-UTI89 were replaced with the promoter regions of *flhDC*, *fliA*, *flhA*, *fliM*, *fliT*, or *fliC*. Thus, the β-galactosidase activities of these resulting strains could reflect the promoter activities of the corresponding flagellar regulon genes. As shown in Figure 4B, in comparison with the strains with *spr* (the WT-UTI89 background), the strains without *spr* (the Δ*spr*-UTI89 background) showed significantly decreased promoter activities of the class 2 (*fliA*, *flhA*, *fliM*, and *fliT*) and class 3 (*fliC*) genes. On the other hand, consistent with the findings regarding the mRNA levels, the strains with and without *spr* showed similar promoter activities of the class 1 *flhDC* genes (Figure 4B). Since these class 2 promoters are specifically regulated by the class 1 master regulator FlhDC (Liu and Matsumura, 1994; Stafford et al., 2005), simultaneous downregulation of these FlhDC-regulated class 2 promoters suggested that FlhDC is involved in the downregulation in the *spr* deletion mutant. In addition, since the transcription of the *flhDC* operon was not interfered by *spr* deletion (Figures 4A,B) and overexpression of *flhDC* increased the motility of the *spr* mutant (Figure 3A), we next investigated whether *spr* deletion affects the intracellular levels of the FlhD and FlhC proteins.

It is known that the levels of endogenous FlhD and FlhC in *E. coli* are too low to be detected by antibodies (Takaya et al., 2006). To determine whether Spr deficiency interferes with the endogenous translation of FlhDC, the *flhDC* genes and their 733 bp upstream uncoding region were PCR amplified from the chromosome of UTI89 and cloned into the high-copy-number plasmid pUC18. In addition, the cloned *flhD* was 5'-end fused with the HA tag sequence and the cloned *flhC* was 3'-end fused with the His_6_ tag sequence. The resulting plasmid was designated pUC18-FlhDC. This plasmid was transferred into ∆*flhDC*-UTI89 and ∆*flhDC*∆*spr*-UTI89. FlhD and FlhC levels in the strains were measured by western blot analysis with the anti-HA and anti-His_6_ antibodies, respectively. As shown in Figure 4C, the strains with and without *spr* showed similar levels of FlhD and FlhC, suggesting that Spr deficiency does not interfere with the translation of FlhDC.

Given that FlhDC-regulated class 2 promoter activities were downregulated and that the intracellular levels of FlhD and FlhC were not affected by *spr* deletion, it is likely that Spr deficiency may decrease bacterial motility by interfering with the formation of the functional FlhDC complex (FlhD_4_C_2_) or by interfering with the efficiency of its gene regulation function.

### Decreased Motility Caused by Loss of *spr* Is Not the Only Defect That Reduces Colonization of the Urinary Tract

**Figure 5:**
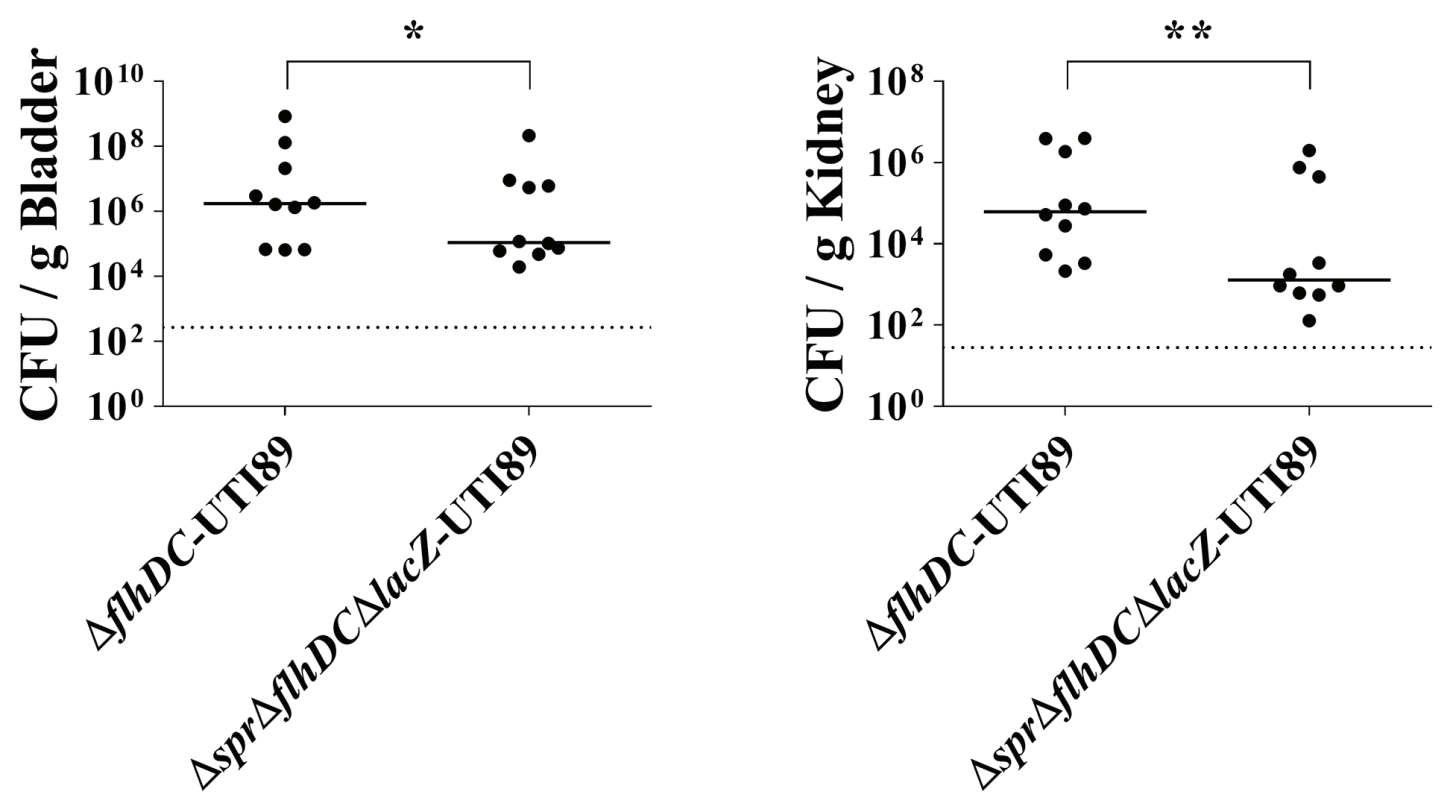
Effect of Spr deficiency on the ability of nonmotile UPEC strains to cause UTIs. The horizontal bars and the dotted lines represent the median levels of the bacterial counts and the limit of detection, respectively. *N* = 10. ^*^ *p* < 0.05; ^**^ *p* < 0.01.

We evaluated whether the reduced motility of the *spr* mutant is the only factor responsible for its competitive fitness during UTIs. We compared the urinary tract colonization abilities of the *flhDC*-deleted UTI89 strains with and without *spr* (∆*flhDC*-UTI89 and ∆*spr*∆*flhDC*∆*lacZ*-UTI89; Table 1). Because of the *flhDC* deletion, the motilities of both strains were abolished (data no shown). After co-inoculation into the mouse urinary tracts for 48 h, ∆*flhDC*-UTI89 exhibited significantly higher bacterial loads in the bladder and kidney than ∆*spr*∆*flhDC*∆*lacZ*-UTI89 (Figure 5). These results demonstrated that under the condition of no bacterial motility, Spr deficiency still reduces UPEC fitness during UTIs, suggesting that there are other Spr deficiency-induced defects that also contribute to decreased fitness of UPEC.

### Deficiency in Spr Impairs Bacterial OM Integrity and Resistance to a Low-pH Environment

**Figure 6:**
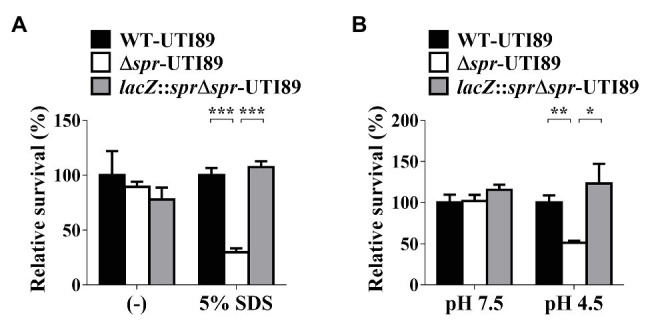
Effect of *spr* deletion on the sensitivity to SDS and low pH conditions. **(A)** The bacterial survival in LB (−) and LB with 5% SDS of the indicated strains was determined after 3 h incubation. **(B)** The survival in LB at pH 7.5 (pH 7.5) and LB at pH 4.5 (pH 4.5) of the indicated strains was determined after 2 h incubation. The levels of bacterial survival are presented as relative survival rates compared to the bacterial counts of WT-UTI89. The results are shown as the means ± standard deviations (SD). ^*^ *p* < 0.05; ^**^ *p* < 0.01; ^***^ *p* < 0.001.

Since *spr* deletion altered the levels of some envelope-associated proteins (Table 2), we investigated whether the alteration compromises the protective function of the envelope structure. The OM is the outermost layer of the envelope and OM integrity is required for bacteria to resist detergent treatment (Ize et al., 2003; Aguiniga et al., 2016). We evaluated the SDS resistance of UTI89 strains with and without *spr*. As shown in Figure 6A, after 3 h of incubation in the media with 5% SDS, the survival of the *spr* deletion mutant (Δ*spr*-UTI89) was significantly lower than those of WT-UTI89 and the complement strain *lacZ*::*spr*Δ*spr*-UTI89. These results suggested that Spr deficiency compromises the protective function of the UPEC envelope against environmental stimuli.

In addition, the bacterial envelope is also the structure providing resistance to environmental acid stresses (Kanjee and Houry, 2013). We investigated whether deletion of *spr* affects UPEC survival in a low-pH environment. As shown in Figure 6B, after incubation in the medium of pH 4.5 for 2 h, the survival of Δ*spr*-UTI89 was significantly lower than those of WT-UTI89 and *lacZ*::*spr*Δ*spr*-UTI89, while in the medium of pH 7.5, these strains showed similar survival levels. These results suggested that Spr deficiency attenuated the protective function of the UPEC envelope to resist low pH.

### *spr* Mutant Exhibits a Lower Ability to Resist Macrophage and Complement System-Mediated Killing

**Figure 7:**
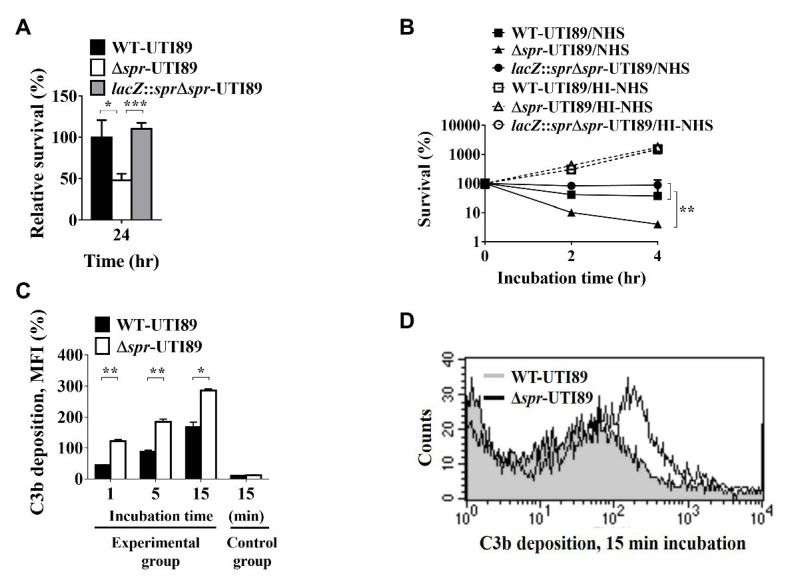
Effect of *spr* deletion on the abilities of UTI89 to survive in macrophages and serum, and the ability to recruit C3b deposition in NHS. **(A)** The intracellular survival of the UTI89 strains with or without *spr* in the macrophage cell line RAW264.7. RAW264.7 cells were independently infected with the indicated UTI89 strains and incubated for 30 min. The infected cells were incubated with 100 μg/ml gentamicin for 15 min to kill extracellular bacteria. Then, the cells harboring intracellular bacteria were incubated with 10 μg/ml gentamicin for the remainder of the experiment. After 0 and 24 h of incubation in the 10 μg/ml gentamicin, the bacterial counts were determined by plating on LB agar plates. The survival rates after 24 h incubation were calculated by the ratio of the bacterial counts after 24 h incubation to those after 0 h incubation. The data are presented as relative survival rates compared to that of WT-UTI89. **(B)** Survival of UTI89 strains with or without *spr* in 30% normal human serum (NHS) or heat-inactivated normal human serum (HI-NHS). The indicated bacteria were independently incubated with 30% NHS or HI-NHS for 2 and 4 h. The levels of bacterial survival are presented as relative survival rates compared to the bacterial counts of the original inoculums. The results are shown as the means ± standard deviations (SD). **(C)** The levels of C3b deposition of WT-UTI89 and Δ*spr*-UTI89 were determined by flow cytometry analysis. WT-UTI89 and Δ*spr*-UTI89 were incubated with 30% NHS for 1, 5, and 15 min (experimental group), and then stained with FITC-conjugated anti-C3 antibody. The bacteria in the control group of the C3b deposition were incubated in PBS instead of 30% NHS for 15 min. The percentage of C3b-labeled bacteria as presented as the mean fluorescence intensity (MFI) are shown as the means ± standard deviations (SD). **(D)** Flow cytometry histogram of C3b deposition on the bacteria after a 15-min incubation in NHS. ^*^ *p* < 0.05; ^**^ *p* < 0.01; ^***^ *p* < 0.001.

To assess whether Spr deficiency interferes with bacterial resistance to phagocyte-mediated intracellular killing. WT-UTI89, Δ*spr*-UTI89, and *lacZ*::*spr*Δ*spr*-UTI89 were independently incubated with macrophage RAW264.7 cells for 30 min. Then, the cultures were treated with a high concentration of gentamicin (100 μg/ml) and incubated for 15 min to kill extracellular (since gentamycin cannot penetrate the membrane of the host cells, the bacteria internalized by the phagocyte are protected from antibiotic killing). Then, the macrophage cells harboring intracellular bacteria were incubated in a lower concentration of gentamycin (10 μg/ml). After 24 h of incubation, the intracellular bacterial loads of Δ*spr*-UTI89 were significantly lower than those of WT-UTI89 and *lacZ*::*spr*∆*spr*-UTI89 (Figure 7A). These results suggested that Spr deficiency decreased the ability of UPEC to survive in phagocytes.

To assess whether deletion of *spr* interferes with bacterial resistance to serum-mediated killing, WT-UTI89, Δ*spr*-UTI89, and *lacZ*::*spr*∆*spr*-UTI89 were independently cultured in normal human serum (NHS) and heat-inactivated human serum (HI-NHS), respectively. After 2 and 4 h of incubation, Δ*spr*-UTI89 and *lacZ*::*spr*∆*spr*-UTI89 exhibited significantly lower survival rates than WT-UTI89 in 30% NHS, while the strains showed similar survival in 30% HI-NHS (Figure 7B). These findings suggested that deletion of *spr* decreased the ability to resist serum-mediated killing. In addition, because in HI-NHS the complement system was inactivated and the strains with and without *spr* showed similar survival in HI-NHS, it is likely that the complement system in NHS was responsible for attenuated serum survival of the *spr* mutant. Since the level of complement component C3b deposition on the bacterial surface could reflect the intensity of the complement activation that occurred on the bacteria in NHS, we measured the level of C3b deposition on the bacteria after incubation in 30% NHS for up to 15 min by flow cytometry analysis. As shown in Figures 7C,D, Δ*spr*-UTI89 showed a higher level of C3b deposition on its surface compared to WT-UTI89, suggesting that deletion of *spr* triggered a higher level of complement activation and thus encountered a stronger complement-mediated attack in NHS than WT-UTI89.

In addition, the antimicrobial peptide cathelicidin LL-37 is an important effector molecular of innate immunity in the urine (Nielsen et al., 2014). We assessed the sensitivities of WT-UTI89 and ∆*spr*-UTI89 to LL-37 and found that deletion of *spr* does not affect UPEC resistance to the antimicrobial peptide (data not shown).

### Deletion of *spr* Reduces Bacterial Numbers in a Bacteremia Co-Infection Model

**Figure 8:**
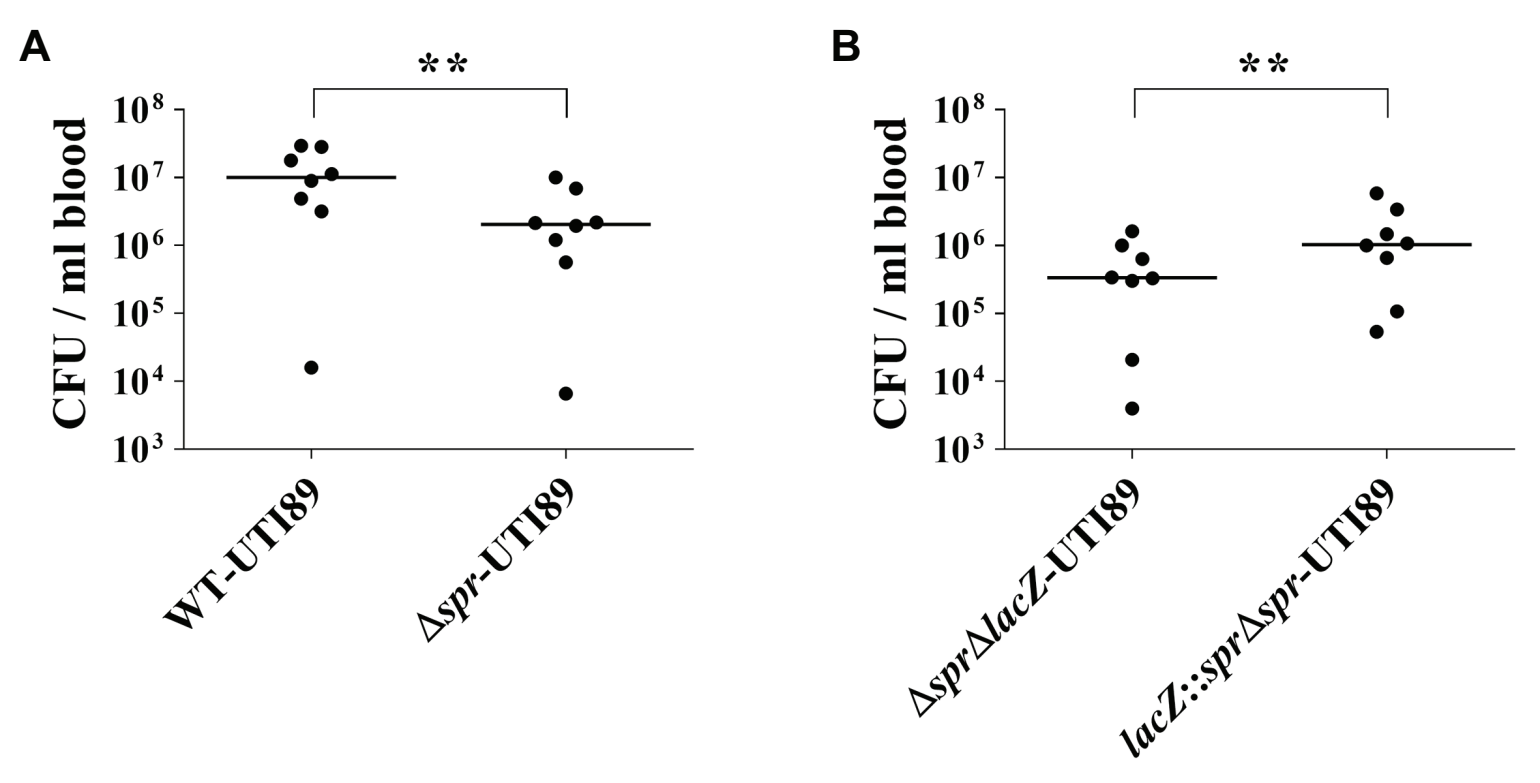
Role of Spr in bloodstream survival UPEC. **(A)** The bacterial blood counts of WT-UTI89 and Δ*spr*-UTI89 in the coinfection mouse model of bacteremia. *N* = 8. **(B)** The bacterial blood counts of the *spr* mutant strain (Δ*spr*Δ*lacZ*-UTI89) and the *spr* complement strain *lacZ*::Δ*spr*-UTI89 in the coinfection mouse model of bacteremia. *N* = 8. The horizontal bars represent the median values. ^**^ *p* < 0.01.

In the cases of descending UTIs, UPEC can invade the urinary tract through the bloodstream, while in some severe cases of ascending UTIs, UPEC in the urinary tract can enter the bloodstream to cause urosepsis. Therefore, the ability of pathogens to survive in the bloodstream is critical for UPEC-caused descending UTIs and urosepsis. The decreased abilities to survive in the serum and to resist phagocyte-mediated killing (Figure 7) implied that Spr deficiency may attenuate UPEC survival in the bloodstream. To test this possibility, equal amounts of UTI89 strains with and without *spr* were co-inoculated into mice through intraperitoneal injection (IP; 5 × 10^6^ CFU/strain/mouse). At 6 h postinoculation, the blood bacterial counts of the strains were determined. As shown in Figure 8A, WT-UTI89 showed significantly higher bacterial counts than Δ*spr*-UTI89. In addition, complementation with *spr* increased the bacterial counts in the bloodstream (Figure 8B). These findings suggested that Spr deficiency reduced the ability of UPEC to survive in the bloodstream.

### Δ*spr*-UTI89 Exhibits a Lower Ability to Switch to Filamentous Morphology After Interaction With Bladder Cells in Comparison With WT-UTI89

**Figure 9:**
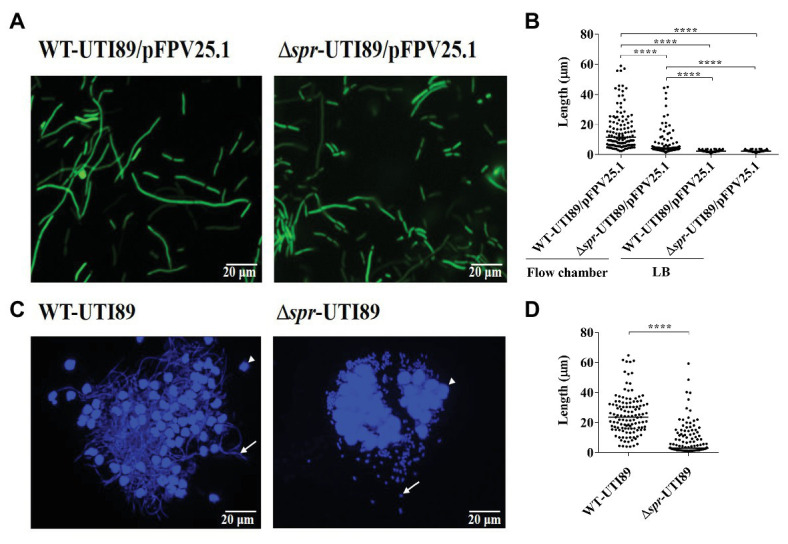
Filamentation of UPEC strains with and without *spr* in the *in vitro* flow chamber-based infection model and the mouse model of UTI. **(A)** Fluorescence microscopic images of WT-UTI89 and ∆*spr*-UTI89 after interaction with bladder epithelial cells in the FC-based infection model of bladder epithelial cells. Both strains harbored the plasmid pFPV25.1 that constitutively expressed GFP. **(B)** The bacterial length of WT-UTI89 and ∆*spr*-UTI89 after incubation in the FC-based infection model and LB medium. For each strain, the length of 120 bacterial cells from three microscopic fields (40 cells/field) was measured. The horizontal bars indicate the median of the bacterial sizes. **(C)** Fluorescence microscopy analyses of UPEC strains recovered from urine at 12 h post infection. The arrows indicates bacteria. The arrow heads indicate sloughed host cells. **(D)** Size quantification of WT-UTI89 or ∆*spr*-UTI89 in the urine samples. The sizes of 120 urine bacteria of each strain from three microscopic fields (40 cells/field) were determined and plotted on the chart. The horizontal bars indicate the median of the bacterial sizes. ^****^ *p* < 0.0001.

It is known that filamentous morphology provides a UPEC advantage to resist phagocytosis (Horvath et al., 2011). We investigated whether Spr deficiency interferes with the switch of UPEC from bacillary to filamentous morphology after interaction with bladder epithelium. To mimic the interaction between UPEC and bladder epithelial cells in UTIs, we utilized an *in vitro* flow chamber (FC)-based bladder cell infection model (Andersen et al., 2012), which allows the morphological transition of UPEC. WT-UTI89 and Δ*spr*-UTI89 were used to infect bladder epithelium monolayers cultured in urine flow chambers (see the Methods section). After co-incubation with the cells, the morphology of the bacteria was investigated by fluorescence microscopy. Elongated filamentous bacteria were found in both the WT and mutant strains (Figure 9A). However, Δ*spr*-UTI89 cells showed a significantly lower level of elongation than WT-UTI89 cells (Figure 9B). On the other hand, in the LB medium, the two strains showed similar bacterial lengths (Figure 9B). These findings suggested that Spr deficiency may attenuate the morphological switch of UPEC in UTIs.

To confirm whether Spr deficiency interferes with the morphological switching of UPEC in UTIs, WT-UTI89 and Δ*spr*-UTI89 were intraurethrally inoculated into mice. At 12 h postinfection, the bacteria in the urine were collected and stained with DAPI. Fluorescence microscopy analysis revealed that a higher level of filamentous bacteria was found in WT-UTI89 than in Δ*spr*-UTI89 cells (Figure 9C). Consistently, size quantification of the bacteria in the urine indicated that the lengths of WT-UTI89 cells were significantly longer than those of Δ*spr*-UTI89 (Figure 9D).

### *spr* Deletion Does Not Affect UPEC Adherence to and Invasion of Uroepithelial Cells

**Figure 10:**
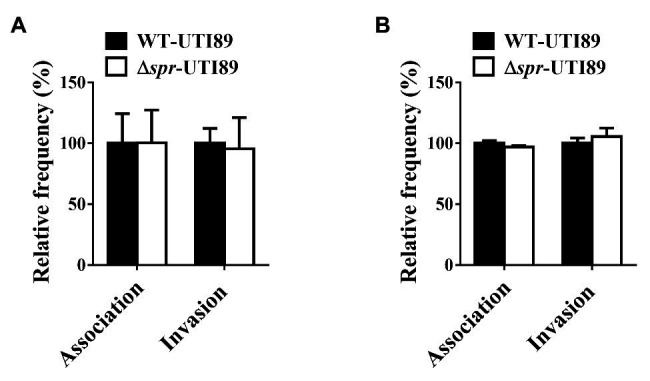
Effect of *spr* deletion on the association and invasion of bladder epithelial cells. The bladder epithelial cells **(A)** T24 cells and **(B)** 5637 cells were incubated with the indicated bacteria. The bars indicate relative association or invasion frequencies compared to the wild-type strain UTI89. The results are shown as the means ± standard deviations.

Adherence to and invasion of uroepithelial cells are critical steps for UPEC to cause UTIs. To determine whether Spr deficiency interferes with the ability of UPEC to interact with uroepithelial cells, the abilities of Δ*spr*-UTI89 and WT-UTI89 to bind to and invade into the bladder epithelial cell lines T24 and 5637 were evaluated. The strains showed similar adherence and invasion rates to the epithelial cell lines (Figures 10A,B), suggesting that Spr deficiency does not affect the ability of UPEC to bind to and invade uroepithelial cells.

## Discussion

This study is the first to demonstrate that the D,D-endopeptidase Spr contributes to the full virulence of UPEC to infect kidneys and is required for optimal fitness to colonize bladders. In addition, the *spr* mutant was significantly outcompeted by the wild-type strain in the bloodstream in the mouse intraperitoneal co-infection model (Figure 8), suggesting that Spr contributes to urosepsis and hematogenous spread of UPEC in the ascending UTIs (hematogenous UTIs). Spr deficiency impaired several virulence properties of UPEC, resulting in attenuation of the infections. Among the impaired properties are reduced motility, compromised competence to evade complement-mediated killing, diminished resistance to intracellular killing by phagocytes, and decreased generation of elongated or filamentous cells during bacterium-uroepithelium interaction. Spr deficiency-induced alteration in the bacterial envelope may be responsible for these impaired virulence properties.

The *E. coli* envelope is a multi-layered structure composed of the IM, PG layer, and OM. The IM and OM enclose the aqueous periplasm in which the PG layer is present. Our LC/MS/MS analysis revealed that the levels of some proteins associated with IM, OM, or periplasm were significantly changed by *spr* deletion, demonstrating that Spr deficiency alters the characteristics of the bacterial envelope (Table 2). These findings also indicate that although Spr is an endopeptidase involved in PG biogenesis (Singh et al., 2012), Spr deficiency alters envelope components other than PG. In addition, the findings that deletion of *spr* increased UPEC sensitivity to detergent treatment and low pH (Figure 6) suggest that the Spr deficiency-induced alteration in the UPEC envelope substantially interfering with the bacterial response and adaptation to environmental stresses (including those within the host).

Flagellum-mediated motility has been suggested to contribute to the dissemination of UPEC, particularly infection of the kidney, during UTIs (Wright et al., 2005; Lane et al., 2007a; Schwan, 2008). Consistently, we found that the reduced motility caused by *spr* deletion was responsible for the decreased urinary tract colonization of the *spr* mutant of UPEC (Figure 3). Spr deficiency reduces UPEC motility by downregulating flagellum expression. The downregulated flagellum expression may be attributed to the altered transcriptional function of the FlhDC master regulator based on the following findings: (i) *spr* deletion downregulated the expression of the FlhDC-governed class 2 genes in the flagellar regulon (Figure 4B) and (ii) the intracellular FlhDC protein levels were not changed by the deletion (Figure 4C). The bacterial factors YdiV and DnaK are known to modify the transcriptional function of FlhDC proteins. DnaK functions as a chaperone to facilitate the formation of the functional transcriptional complex, while YdiV reduces the interaction between the FlhDC complex and the promoter DNA (Takaya et al., 2006, 2012). However, we found that the protein levels of DnaK are similar in WT-UTI89 and ∆*spr*-UTI89 (Supplementary Figure S3) and that in the *ydiV* deletion background, *spr* deletion still significantly downregulated UPEC motility (Supplementary Figure S4). The findings suggested that DnaK and YdiV are not involved in Spr deficiency-induced flagellum downregulation and that a currently unrevealed mechanism to modify FlhDC function exists. It is likely that that *spr* deletion interferes with the efficiency of the formation of functional FlhDC complexes (FlhD_4_C_2_) or interferes with the functional efficiency of the complex in gene regulation, leading to decreased flagellum expression and motility reduction. This hypothesis may explain why overexpressing FlhD and FlhC increases the motility of the *spr* mutant (Figure 3A). The high levels of FlhD and FlhC may facilitate the formation of the functional FlhDC complex and increase its intracellular concentration in the *spr* mutant, thus increasing the bacterial motility.

In the co-infection experiments, the bacterial counts of the *spr* mutant with pUC19 (Δ*spr*Δ*lacZ*-UTI89/pUC19; Figure 3B) were quite low in comparison with those of the *spr* mutant without the plasmid (Figure 1A,B). This may be due to a fitness burden imposed by the pUC19. It is known that high copy number plasmids impose metabolic burden in *E. coli* (Togna et al., 1993; Silva et al., 2012). Given that pUC19 is a high copy number plasmid and *spr* deletion interferes with bacterial physiology (Hara et al., 1996; Vesto et al., 2018), the plasmid may induce a substantial fitness burden in the *spr* mutant in bladders and kidneys. In the same co-infection experiment (Figure 3B), the competing strain was a *spr* mutant with the pUC19-derived pFlhDC (Δ*spr*-UTI89/pFlhDC). The improved motility induced by overexpressing FlhD and FlhC may increase competitive fitness to a level high enough to overcome the fitness burden induced by pFlhDC during UTIs. Therefore, Δ*spr*Δ*lacZ*-UTI89/pUC19 was significantly outcompeted by Δ*spr*-UTI89/pFlhDC, resulting in such a low bacterial burden.

The *spr* mutant of UPEC showed decreased bacterial counts that the wild-type in the co-infection model of bacteremia (Figure 8). The reduced survival may be at least partially due to the mutant’s decreased ability to evade the complement-mediated attack (Figures 7D–F), since the complement system is one of the major branches of host innate immunity against invading pathogens in the bloodstream (Sarma and Ward, 2011). Previous studies have shown that the complement system is functional in urinary tracts, suggesting that the system may contribute to pathogen clearance in urinary tracts (Brooimans et al., 1991; Seelen et al., 1993; Song et al., 1998; Li et al., 2006, 2008). However, it has also been shown that opsonization of UPEC with the C3 complement component facilitates UPEC invasion of uroepithelial cells and that mice deficient in C3 are resistant to ascending UTI of UPEC. These findings suggest that the final outcome of the interaction between UPEC and the complement systems in urinary tracts may facilitate infection. Therefore, it remains to be investigated whether the enhanced complement attack caused by *spr* deletion facilitates or hinders pathogen infection in urinary tracts, although the overall effect of *spr* deletion reduce the competitive fitness of the strain compared to the wild-type in the urinary tract (Figure 1).

The compromised OM integrity may be responsible for the reduced ability of the *spr* mutant to evade complement-mediated attack. OM is where the invading bacteria interact with the complement system in the hosts (Miajlovic and Smith, 2014). Previous studies have shown that impairing the OM integrity of pathogenic *E. coli* through inactivation of envelope-associated bacterial factors, such as OM proteins OmpA and NlpI, and the periplasmic protease Prc, induces stronger complement-mediated attack in NHS (Wooster et al., 2006; Tseng et al., 2012; Wang et al., 2012; Miajlovic and Smith, 2014). Therefore, the Spr deficiency-induced interference of OM integrity may trigger higher levels of complement-mediated attack, and thus attenuate serum survival of the *spr* mutant. In addition, a high-throughput transposon mutagenesis-based study with an UPEC ST131 strain has identified 41 genes that contribute to bacterial serum survival (Phan et al., 2013). The majority of them encode membrane proteins and factors involved in LPS biogenesis. Mutants of 23 (56%) of these genes show increased sensitivities to SDS, suggesting that inactivation of these genes also compromise the OM integrity. Although the mechanisms by which these genes contributes to serum resistance are not elucidated yet, the defected OM integrity caused by the deletion of these genes may attenuate the bacterial ability to evade the complement-mediated attack, like the defect caused by the *spr* deletion, and thus decreases bacterial survival in the serum.

Spr deficiency reduces UPEC resistance to phagocyte-mediated killing in two distinct manners. The deficiency attenuated UPEC intracellular survival in phagocytes (Figure 7A) and reduced the UPEC morphological switch to filamentous shapes. Filamentation can prevent engulfment by phagocytes (Horvath et al., 2011). The defective intracellular survival in phagocytes may contribute to the *spr* mutant’s decreased survival in the bloodstream and the urinary tracts.

After internalization by phagocytes, bacterial pathogens are constricted and killed within phagolysosomes, where a low-pH environment contains degradative enzymes (Jabado et al., 2000; Pillay et al., 2002). Thus, the impaired OM and the downregulated low-pH tolerance may damage the *spr* mutant resistance to enzyme-mediated degradation and the low-pH environment.

The UPEC morphological switch to filamentous shapes results from the prevention of cell division (Khandige et al., 2016). Spr deficiency may relieve division inhibition in UPEC during the course of UTIs. *Escherichia coli* employs two-component systems to monitor damaged bacterial envelope (Bury-Mone et al., 2009). In addition, the activation of an *E. coli* two component system RcsCDB increases the expression of cell division factors FtsZ and FtsA (Carballes et al., 1999). It is known that a high level of FtsZ is able to resist cell division inhibitor MinCD in *E. coli* (Ward and Lutkenhaus, 1985). Although the levels of RcsCDB activation in WT-UTI89 and Δ*spr*-UTI89 were similar (data not shown), it is likely that the interfered envelope structures induced by Spr deficiency may trigger the activation of certain signaling system (s) other than RcsCDB to relieve the inhibition of cell division during the course of UTIs. This possibility warrants further investigation.

Spr (MepS) and the other two murine endopeptidases, YebA (MepM) and YdhO (MepH), are proposed to be functionally redundant in *E. coli* growth, because a triple mutant of these enzymes fails to grow in LB medium, while complementation with any of these genes confers growth (Singh et al., 2012). Similarly, the present study showed that Spr deletion did not interfere with the growth of UPEC in LB and M9 media, and even urine at 37°C (Supplementary Figure S2). However, the function of Spr may not be completely redundant with that of YebA and YdhO. For examples, it has been reported that *spr* deletion, despite YebA and YdhO are present, distinctly induces a grow defect of *E. coli* under combined conditions of high temperature and low osmolality (Hara et al., 1996). In the present study, loss of *spr* alone induced *in vitro* and *in vivo* phenotypes relating to virulence properties. These findings indicate that the presence of YebA and YdhO functions cannot cover the loss of Spr function, suggesting that the role Spr is distinct from YebA and YdhO in certain conditions. Accordingly, we speculate that the *spr* deletion-induced phenotypes may not necessarily be present in the *yebA* or *ydhO* mutant although they exhibit a similar endopeptidase function. Consistent with this speculation, it has been reported that deletion of *spr* impairs intrinsic vancomycin resistance in *S. enterica* serovar Typhimurium but deletion of *yebA* or *ydhO* does not (Vesto et al., 2018). Therefore, it would be worthy to extend the investigation on the roles of *yebA* and *ydhO* in *E. coli* infections.

Type-1 fimbia is a well-known adherence factor of UPEC to bind to bladder epithelial cells during UTIs (Kaper et al., 2004). It has been reported that in a neonatal meningitides *E. coli* strain RS218 *spr* deletion increases the ratio of the colonies containing Type 1 fimbria phase-ON cells when they are grown on agar plates, and that the crosstalk between the expression of Type 1 fimbria and flagella has been shown in *E. coli* strains (Barnich et al., 2003; Lane et al., 2007b; Chen et al., 2014). However, Spr deficiency may not affect the expression of Type 1 fimbria in the UPEC strain UTI89, because WT-UTI89 and ∆*spr*-UTI89 showed similar levels of Type 1 fimbria phase-ON bacteria in the culture (Supplementary Figure S5). In agreement with this finding, the WT and *spr* mutant strains showed a similar ability to bind to bladder epithelial cells (Figure 10). Therefore Type 1 fimbria function may not be involved in the defected fitness and attenuated virulence in the UTI89 *spr* mutant during UTIs.

In summary, in the present study, we demonstrated that inactivation of the PG endopeptidase Spr decreases the abilities of UPEC in urinary tract colonization and bloodstream survival. The Spr deficiency-induced downregulation in bacterial motility, resistance to phagocyte- and complement-mediated attacks, and reduced morphological plasticity. The broad range of impacts of Spr inactivation on the virulence properties of UPEC indicates that Spr is a potential target for developing novel antimicrobial strategies in UTIs.

## Data Availability Statement

The original contributions presented in the study are included in the article/Supplementary Material, further inquiries can be directed to the corresponding author.

## Ethics Statement

The studies involving human participants were reviewed and approved by The Institutional Reviewer Board (IRB) of National Cheng Kung University Hospital, Tainan City, Taiwan (no. ER-98-143 and B-ER-108-308). The patients/participants provided their written informed consent to participate in this study. The animal study was reviewed and approved by The Institutional Animal Care and Use Committee (IACUC) of National Cheng Kung University, Tainan City, Taiwan (approval number. 105175 and 107175).

## Author Contributions

W-CH carried out the experiments in this study. W-CH, MH, and C-HT contributed to the study conception, planning experiments, data analysis, and interpretation. C-CW participated in the technical support of Flow chamber-based cell infection model. M-FL and Y-LC contributed the materials and technical supports of the animal experiments. Y-LS and J-JW participated in conceptualization and interpretation of the data. M-CW, W-HL, and M-YH contributed materials and technical support. W-CH and C-HT wrote the manuscript. All authors contributed to the article and approved the submitted version.

### Conflict of Interest

The authors declare that the research was conducted in the absence of any commercial or financial relationships that could be construed as a potential conflict of interest.
