# Supplementary material for: Peptidoglycan Endopeptidase Spr of Uropathogenic Escherichia coli Contributes to Kidney Infections and Competitive Fitness During Bladder Colonization
Source: Front Microbiol. 2020 Dec 16;11:586214. doi: 10.3389/fmicb.2020.586214 (PMC7774453; doi:10.3389/fmicb.2020.586214)
Supplement: Supplementary file 1 [file Data_Sheet_1.pdf]

Supplementary Figure S1

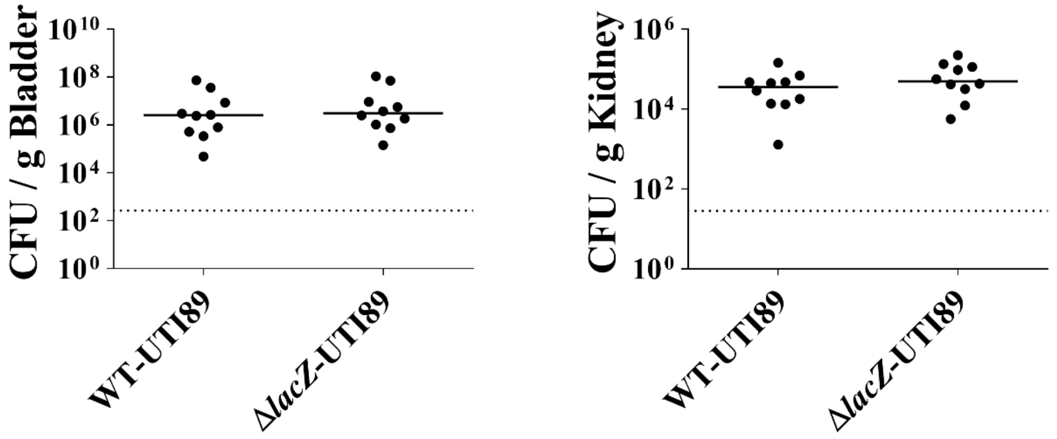

Supplementary Figure S1. Deletion of *lacZ* did not affect the ability of UPEC to cause UTIs. The bacterial counts of WT-UTI89 and Δ*lacZ*-UTI89 in the bladders and kidneys at 48 h after transurethral coinoculation with equal amounts of these strains ($5x10^7$ CFU/mouse for each strain). Animal numbers (N) =10.

Supplementary Figure S2

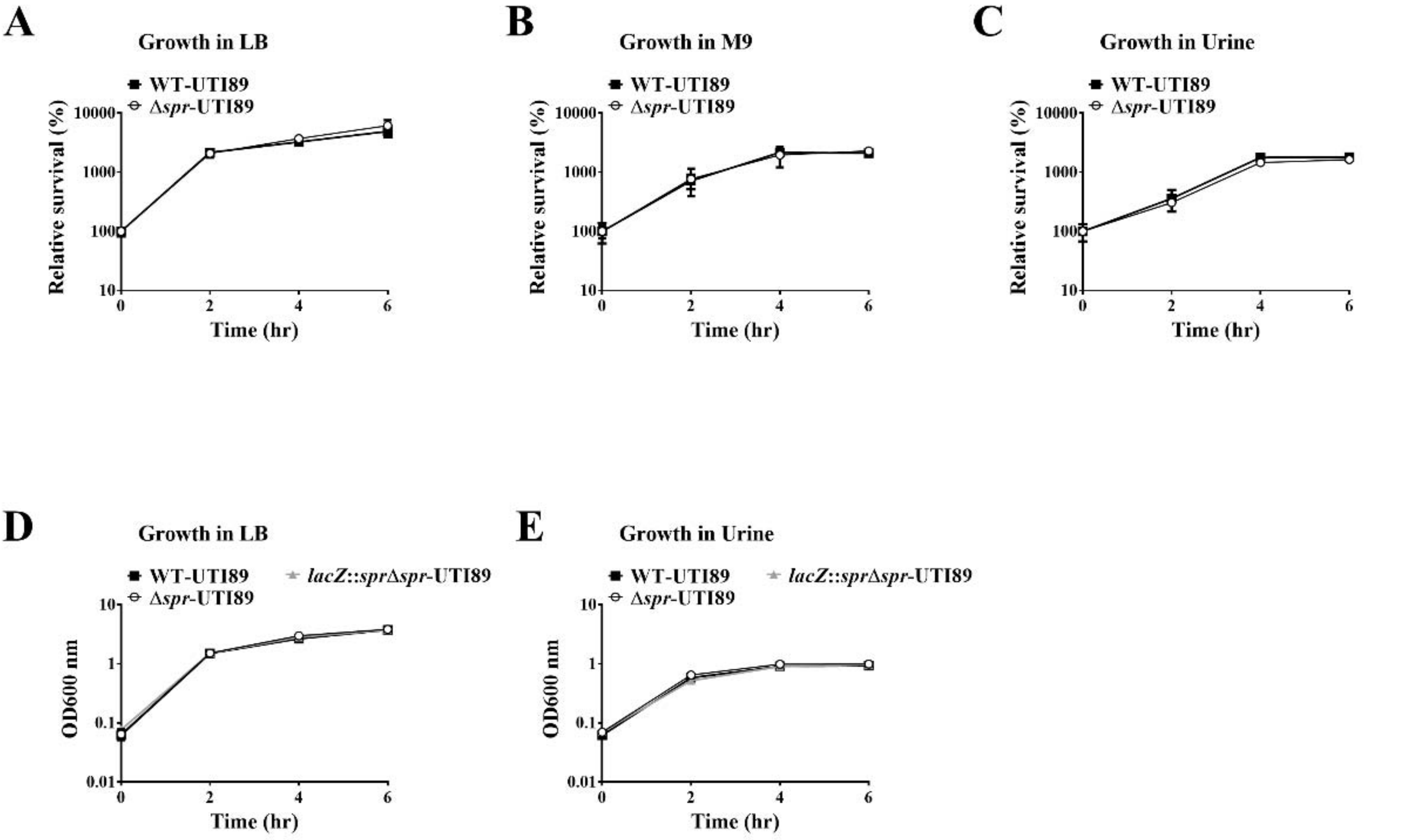

Supplementary Figure S2. The growth curves of UIT89 strains in different media. (A), (B), and (C) WT-UTI89 and Δ*spr*-UTI89 were co-cultured in LB broth (A), M9 medium (B), and urine (C). Equal numbers of the bacterial strains (1×10^6 CFU/strain) were inoculated in 100 µl of fresh media and incubated at 37°C. The bacterial counts were determined by plating the culture on LB agar plates with and without chloramphenicol to differentiate the wild type (chloramphenicol sensitive) and the *spr* mutant (chloramphenicol resistant). The growth curves were presented as relative growth rates compared to those at 0 h of incubation. (D) and (E) The independent growth curves of the indicated UIT89 strains in LB broth (D), and urine (E). 16-h bacterial cultures were diluted 1:100 in 5 ml LB broth or urine, and incubated at 37°C. The growth of the strains was determined by measuring the values of O.D.600 at the indicated time points using a spectrophotometer. Data are shown as the mean ± SD of three independent experiments.

Supplementary Figure S3

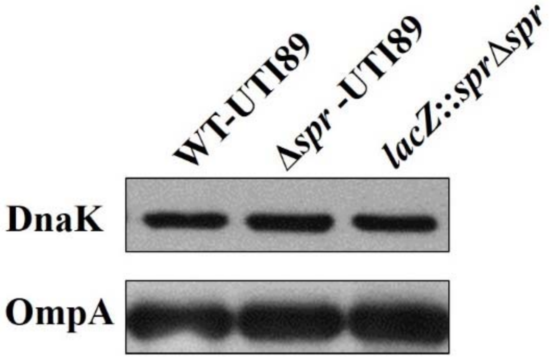

Supplementary Figure S3. Deletion of *spr* did not affect the levels of DnaK.

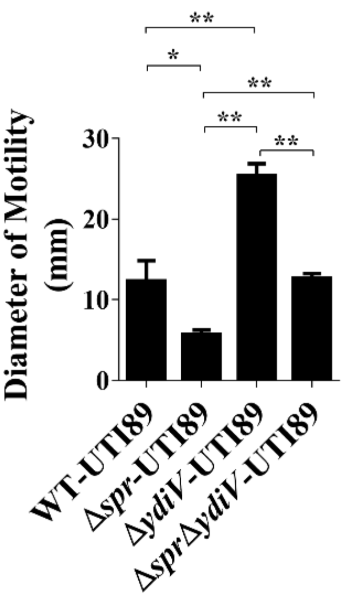

Supplementary Figure S4. Deletion of *spr* still decreased motility in the *ydiV* deletion background.

Supplementary Figure S5

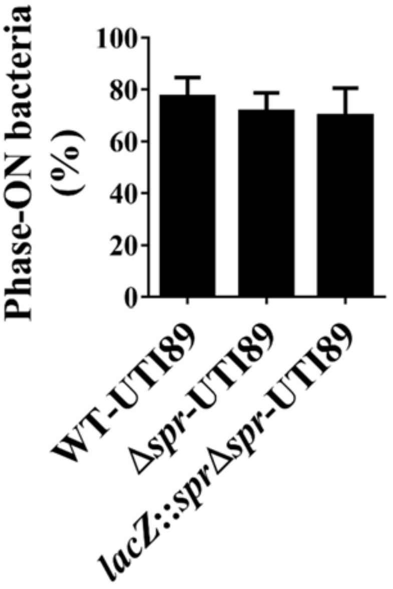

Supplementary Figure S5. The ratios of type 1 fimbria Phase-ON bacteria in WT-UTI89, Δ*spr*-UTI89, and *lacZ*::*spr*Δ*spr*-UTI89. Bacteria from 16-h cultures in LB medium were subjected to invertible element orientations assay as described previously (Chen et al., 2014) to determine the ratios of the type 1 fimbria Phase-ON bacteria in the cultures of the three strains. Data are shown as the mean ± SD of experiments in triplicate. There were no significant difference in the Phase-ON ratios among the cultures of the three strains, suggesting that deletion of *spr* does not affect type 1 fimbria expression.

Reference

Chen, Y.W., Teng, C.H., Ho, Y.H., Jessica Ho, T.Y., Huang, W.C., Hashimoto, M., Chiang, I.Y., and Chen, C.S. (2014). Identification of bacterial factors involved in type 1 fimbria expression using an Escherichia coli K12 proteome chip. *Mol Cell Proteomics* 13**,** 1485-1494.

Supplementary Table S1. Primers used in this study.

Primers	Sequence (5'→3')
**Gene knockout**	
NK-*lacZ*-F	GTCGTGACTGGGAAAACCCTGGCGTTACCC AACTTAATCGCATATCAATATCCTCCTTAG
NK-*lacZ*-R	CAACTGGTAATGGTAGCGACCGGCGCTCAG CTGGAATTCCGTGTAGGCTGGAGCTGCTTC
NK-*spr*-F	TTGTCGTTAAGGACTTCAAGGGAAAACAA ACAACATGGTCCATATGAATATCCTCCTTAG
NK-*spr*-R	GAGAACCCGGCGTGCTTCGTTGTAACGCTT CTTCCAGTATGTGTAGGCTGGAGCTGCTTC
NK-*flhDC*-F	AGAAATGGCGACAACGTTAGCGGCACTGA CTCTTCCGCAAATAGGAATATCCTCCTTAG T
NK-*flhDC*-R	GTGGGATAATATCGGCAGGATTCTGGGAA AGTTTACGTCTTGTGTAGGCTGGAGCTGCT T
**Complementary *spr gene* at the *lacZ* gene chromosomal locus**	
The upstream region	
FD-lacZ-F	CCGGAAGAGAGTCAATTCAG
LacI-500-R	CATATGATAAGCATCGCTGCTCATACCAAAG CCGTTGATGGGTGTCTGGTCAG
The *spr*-3xFlag-Cm region	
lacZ-spr-F	CTTGATGTCTCTGACCAGACACCCATCAAC GTTGCTGACGCCATTAAGTG
New P1	TGTGTAGGCTGGAGCTGCTT
The downstream region	
LacZ down 500-F	AAGCAGCTCCAGCCTACACAGAGCTCCTGC ACTGGATGGTG
141-30-2	AGACCAACTGGTAATGGTAG
**The *spr* point mutation strain (Spr-C68A)**	
The upstream region of C68A	
Spr promoter-F	GTTGCTGACGCCATTAAGTG
Spr-C68A-R	GAAACCAGACGCATCGATACCTTTTTAGT GC
The downstream region of C68A	
Spr-C68A-F	GTATCGATGCGTCTGGTTTCGTACAGCGTAC
spr500-R	GTAAGATCGCCATATCGACG

**The flagellar genes' promoter-lacZ fusion strains**	
The upstream region	
check-no lacI-F	GCTGTGCAACACAATACTGC
no lacI-UP500-R	TGACGCCGAAGTGAGATTTAAAATGCTGAC
The chloramphenicol-resistance cassette	
no lacIp-Cm-F	GACCTGGCGTCAGCATTTTAAATCTCACTTCGGCGTCATGTGTAGGCTGGAGCTGCTTCG
NEW-P2	ATAGGAATATCCTCCTTAGTTC
flhD-lacZ	
Cm-flhDp-F	CGGAATAGGAACTAAGGAGGATATTCCTATTGATGTGTCCCTTTACTGGC
Cm-flhDp-R	TCCTGTGTGAAATTGTTATTCGCTCACAATTTTATGCGGTCTCACCGCAC
fliA-lacZ	
Cm-fliAp-F	CGGAATAGGAACTAAGGAGGATATTCCTATCGACCGAAGTGTCCAACATG
Cm-fliAp-R	TCCTGTGTGAAATTGTTATTCGCTCACAATCGGCATGATTATCCGTTTCTGC
flhA-lacZ	
Cm-flhAp-F	CGGAATAGGAACTAAGGAGGATATTCCTATGTCAGTTGCTGATGGTGCTG
Cm-flhAp-R	CTGTGTGAAATTGTTATTCGCTCACAATAAGCGTAAATGATGCCAGAG
fliM-lacZ	
Cm-fliMp-F	CGGAATAGGAACTAAGGAGGATATTCCTATACACCAGACACAGCCACTGC
Cm-fliMp-R	CTGTGTGAAATTGTTATTCGCTCACAATCGGCTAATTATCCTGCGTCTTG
fliT-lacZ	
Cm-fliTp-F	CGGAATAGGAACTAAGGAGGATATTCCTATATACGCAAGCCAGAAGACAG
Cm-fliTp-R	CTGTGTGAAATTGTTATTCGCTCACAATGAATTGCGCAAAGTTTACG
fliC-lacZ	
Cm-fliCp-F	CGGAATAGGAACTAAGGAGGATATTCCTATGATGGGTGACGCTGATGGTG
Cm-fliCp-R	TCCTGTGTGAAATTGTTATTCGCTCACAATA

	ACCCTGTTATCGTCTGTCG
The downstream region	
lacZ-500-F	ATTGTGAGCGAATAACAATTTCACACAG
lacZ-500-R	ACCACAGATGAAACGCCGAG
**qPCR**	
gyrB-RT-F	CTATAAAGTGTCCGGCGGTC
gyrB-RT-R	CGGAGTTGAGGAACGACAAC
flhD-RT-F	TCCGCTATGTTTCGTCTCGGCATA
flhD-RT-R	ACCAGTTGATTGGTTTCTGCCAGC
fliA-RT-F	AACGCTATGACGCCCTACAAGGAA
fliA-RT-R	AGTTCCTGCTCCAGTTGCCCTATT
flgE-RT-F	CACGTTTAGCCTGAGCTTCC
flgE-RT-R	CAACCGTACCGTCATCATTG
flhA-RT-F	ACGAGAAACCGACCCATGAG
flhA-RT-R	CCATCATCGACAAGATCAAC
fliF-RT-F	AATGCGACTGCAGCCCAGAC
fliF-RT-R	AGGATCAGTGCGACCATGAC
fliM-RT-F	GATAACGACATGGGCGATAG
fliM-RT-R	CACTTTCGCCGCTAACACTG
fliE-RT-F	GTTATCAGCCAGTTACAGGC
fliE-RT-R	TGTGTATCGCTTATGCGATC
fliT-RT-F	ATTTCGCCTGGCAACAACTC
fliT-RT-R	TGCACCGCATTCACATACGC
flgM-RT-F	GAGTATTGATCGCACTTCGC
flgM-RT-R	ACGTCACACTGGTGCTGGTG
fliC-RT-F	ACAGCCTCTCGCTGATCACTCAA
fliC-RT-R	GCGCTGTTAATACGCAAGCCAGA
motA-RT-F	GCGATTAAAGGCACGCTGAAGG
motA-RT-R	GAAGGTGTTCATGTGACCGCTG
**Plasmid pUC18-FlhDC**	
flhDC promoter-F	GCGATAGATACCGCTTTTGCCAGCAGTTGC
flhDC promoter-R	TCCAGCATAATCTGGAACATCATATGGATA
	CACCCAGAATAACCAACTTTATTTTTATGC
HA-*flhD*-F	TATCCATATGATGTTCCAGATTATGCTGGA
	ATAATGCATACCTCCGAG
flhC-R	CCGGGGATCCTTAATGATGATGATGATGAT
	GAACAGCCTGTACTCTCTGTTC
